# CircSpna2 attenuates cuproptosis by mediating ubiquitin ligase Keap1 to regulate the Nrf2‐Atp7b signalling axis in depression after traumatic brain injury in a mouse model

**DOI:** 10.1002/ctm2.70100

**Published:** 2024-11-24

**Authors:** Mengran Du, Jiayuanyuan Fu, Jie Zhang, Ziyu Zhu, Xuekang Huang, Weilin Tan, Lian Liu, Zhijian Huang, Xin Liu, Qiuhao Tan, ZhengBu Liao, Yuan Cheng

**Affiliations:** ^1^ Department of Neurosurgery The Second Affiliated Hospital of Chongqing Medical University Chongqing China; ^2^ Department of Neurosurgery The First Affiliated Hospital of Chongqing Medical University Chongqing China

**Keywords:** circRNAs, cuproptosis, depression, traumatic brain injury, ubiquitination

## Abstract

**Background:**

Depression is a common but often overlooked consequence in individuals with post‐traumatic brain injury (TBI). Circular RNAs (circRNAs) play essential roles in the nervous system, yet their involvement in the cell death mechanism known as cuproptosis and in TBI‐related depression remains unclear.

**Objectives:**

This study aimed to investigate the role of circRNA, specifically circSpna2, in the regulation of cuproptosis and its association with depression in TBI patients.

**Methods:**

RNA sequencing (RNA‐Seq) was used to assess the differential expression of circRNAs. Depression was evaluated using subjective and objective rating scales, and circSpna2 expression levels in plasma were measured. Further functional experiments were conducted in TBI mouse models, including knockdown and overexpression of circSpna2, to explore its impact on the Keap1‐Nrf2‐Atp7b pathway and cuproptosis.

**Results:**

TBI patients exhibited decreased levels of circSpna2, which correlated with depression (*p* < 0.0001). Knocking down circSpna2 in TBI mice aggravated depression‐like symptoms (*p* < 0.0001). Mechanistically, circSpna2 was found to bind ubiquitin ligase Keap1, modulating the Nrf2‐Atp7b signaling pathway and influencing cuproptosis (docking score: −331.88). Overexpression of circSpna2 alleviated cuproptosis after TBI through the Keap1/Nrf2/Atp7b axis.

**Conclusions:**

CircSpna2 plays a regulatory role in cuproptosis and may serve as a novel biomarker and therapeutic target for depression following TBI. Enhancing circSpna2 expression could mitigate depression after TBI by modulating the Keap1/Nrf2/Atp7b pathway.

**Key points:**

This study explores the role of circSpna2 in depression following traumatic brain injury (TBI). It was found that circSpna2 is significantly downregulated in TBI patients, and its expression levels correlate with depressive symptoms. In TBI mouse models, overexpression of circSpna2 alleviated depression‐like behaviours, while its knockdown exacerbated these symptoms, suggesting its potential as both a biomarker and a therapeutic target for post‐TBI depression. Mechanistically, circSpna2 regulates the Nrf2‐Atp7b signalling pathway by binding to the DGR domain of Keap1, which prevents Nrf2 ubiquitination and enhances Nrf2 activity. This in turn promotes the transcription of Atp7b, a copper transport protein, helping to maintain copper homeostasis and mitigate copper‐induced oxidative stress, a key driver of cell death (cuproptosis). The overexpression of circSpna2 also improved mitochondrial function and synaptic integrity, which are typically impaired by copper dysregulation. These findings highlight the therapeutic potential of circSpna2 in managing TBI‐related depression through the regulation of oxidative stress and copper homeostasis.

## Graphical abstract

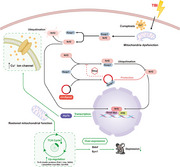

## 1 INTRODUCTION

Depression frequently accompanies traumatic brain injury (TBI) and can manifest anytime within the first 6 months post‐injury.^1^ TBI also increases the incidence rate of chronic emotional damage, such as persistent anxiety, depression and mood swings. Both TBI and post‐TBI depression seriously affect patients’ daily lives, reducing the quality of life and increasing social burden.^2^ Research has consistently shown that individuals with TBI experience higher rates of depression than the general population, affecting nearly half of these patients.^3^ Statin medications like simvastatin^4^ and lovastatin^5^ were reported to attenuate neuroinflammation to reduce depression after TBI. Other drugs, such as oxyberberine,^6^ buprenorphine,^7^ and sertraline,^8^ were reported to have protective effects on psychiatric disorders after TBI. Although post‐TBI depression is often manifested, effective therapies, precise mechanisms and molecular interactions have not been identified.

TBI frequently causes major disturbances in copper homeostasis, resulting in both immediate and long‐term impacts on neurological function. In acute copper dyshomeostasis, oxidative stress is intensified, causing an overproduction of reactive oxygen species (ROS) and subsequent neuronal damage. This oxidative stress can impair mitochondrial function, further contributing to cell death and acute neuronal injury.^9^, ^10^ The chronic effects of copper imbalance post‐TBI include sustained inflammation and ongoing neurotoxicity, which can hinder neurogenesis and synaptic plasticity. Over time, these changes can result in long‐term cognitive deficits, mood disorders and heightened susceptibility to neurodegenerative conditions.^11^, ^12^ Dysregulated copper levels can also affect key signalling pathways and metabolic processes critical for maintaining neuronal health.^13^ Understanding the mechanisms by which copper dyshomeostasis impacts the brain is crucial for developing targeted therapeutic strategies. Addressing copper imbalances may offer a novel approach to mitigating both the immediate and prolonged consequences of TBI, potentially improving outcomes for affected individuals.^14^, ^15^

Cuproptosis represents a type of cell death controlled by a unique regulatory mechanism involving copper.^16^ Copper (Cu), a transition metal, is crucial in numerous biological functions and vital for preserving cellular equilibrium. An imbalance can lead to oxidative stress and other pathological conditions. Research indicates that elevated copper levels can trigger depression‐like symptoms and other neurological disorders.^10^ Copper is a vital microelement affecting gene expression alterations, biological metabolic processes and various signalling pathways.^17^ Many recent studies have highlighted the roles of cuproptosis and copper metabolism in central nervous system disorders, including Parkinson's disease (PD),^11^ Alzheimer's disease (AD)^12^ and cerebral ischaemia–reperfusion injury.^18^ Lei et al. identified a cuproptosis‐related gene in humans with major depressive disorder,^19^ suggesting that cuproptosis might be involved in depression symptoms. Some studies have reported a relationship between cuproptosis and TBI, and copper homeostasis dysfunction after brain injury can have numerous acute and chronic effects on neurological function.^20^ However, a research gap on the role of cuproptosis in post‐TBI depression exists.

Circular RNAs (circRNAs), categorised as non‐coding RNAs (ncRNAs), are implicated in various diseases and play an essential role in numerous pathophysiological processes by interacting with RNAs and proteins.^21^, ^22^ CircRNAs are known to be differentially expressed after TBI.^23^ In a previous study, we reported that circLphn3^24^ played a protective role against blood–brain barrier impairment following TBI and identified an altered expression profile of ncRNAs after melatonin treatment post‐TBI.^25^ Recent studies have highlighted the diverse roles of circRNAs in regulating various cellular processes and disease mechanisms. For example, circPtpn14 has been identified as a regulator of ferroptosis, a form of cell death triggered by iron‐catalysed lipid peroxidation. This process is implicated in numerous diseases, including cancer and neurodegeneration. Understanding the role of circRNAs in ferroptosis can provide critical insight into their potential as therapeutic targets. In the context of TBI, we hypothesise that circRNAs, such as circSpna2, may influence similar pathways, contributing to the complex pathology of TBI.^26^ However, studies illustrating the function of circRNAs in post‐TBI depression are lacking. Several studies have described the function of ncRNAs in depression. Zou et al. investigated candidate ncRNAs in major depressive disorder pathogenesis,^27^ while Yu et al. reported that circDYM delivered by extracellular vesicles attenuated chronic unpredictable stress‐induced depression.^28^ Another study found that 2210408F21Rik influenced depressive‐like behaviours by regulating the miR‐1968‐5p/Hras axis.^29^ Thus, the role of circRNAs in post‐TBI depression, as well as the potential regulatory role of cuproptosis, warrants further investigation.

The present study used the Hamilton Depression Scale (HAMD), Montgomery–Åsberg Depression Rating Scale (MADRS), self‐rating depression scale (SDS) and Beck Depression Inventory (BDI) to identify depression disorders in patients with TBI. We also found that circSpna2 was linked to post‐TBI depression symptoms in these patients. Finally, the experiments in vivo and in vitro were performed to verify the functional role of circSpna2, identifying that the regulatory mechanism is the Keap1/Nrf2/Atp7b signalling axis.

## 2 METHODS

### 2.1 Preparation of human brain specimens

Contused brain tissue samples (approximately 1 cm^3^) were collected from six patients undergoing craniotomy 5–20 h after severe TBI. Additionally, three control samples were surgically collected from the cerebral cortex for World Health Organization Grade I tumours located in the ventricles (Supporting Information Table 1). Brain tissue samples were stored in liquid nitrogen during surgery and transferred to −80°C for subsequent analysis after surgery.

### 2.2 Patient recruitment and information

TBI patients were selected from the Neurosurgery Department at the First Affiliated Hospital of Chongqing Medical University between May 2023 and April 2024. A total of 60 TBI patients and 40 healthy controls were recruited. The criteria for TBI patient inclusion were: (1) age 18–65 years, (2) experienced TBI within the past year, (3) Glasgow Coma Scale scores (GCSs) of 8–13 before admission and (4) the provision of informed consent. The exclusion criteria were: (1) previous history of brain injury, cerebral surgery or other craniocerebral diseases; (2) medical history of psychiatric diseases; (3) currently pregnant; (4) multiple organ failure or other serious systemic complications; (5) hospitalisation time >1 month; and (6) dropped out of the study. All clinical data are shown in Supporting Information Table 2.

### 2.3 Plasma collection

Human plasma samples were obtained 90 days post‐TBI, a period selected to observe the development and continuity of depressive symptoms.^30^, ^31^ Mouse plasma samples were obtained 1, 3, 7, 15 and 30 days after TBI. Blood samples were drawn into EDTA tubes and promptly chilled on ice. The samples were centrifuged and collected the plasma, aliquoted and stored at −80°C for later analysis. The plasma samples were analysed using polymerase chain reaction (PCR) to investigate circRNA expression changes associated with TBI and depression.

### 2.4 Diagnosis of depression in TBI patients

The diagnosis of depression encompasses but is not restricted to, the following symptoms persisting for 2 weeks or more: (1) loss of interest; (2) fatigue; (3) psychomotor changes; (4) self‐esteem issues or guilt; (5) suicidal thoughts or self‐harm; and (6) sleep disturbances, like insomnia or oversleeping.^32^ This study utilised the HAMD, MADRS, SDS and BDI to evaluate depressive symptoms in TBI patients. HAMD scores of >20, MADRS scores of >11, SDS scores of >52 and BDI scores of >15 were indicative of depression disorders.

### 2.5 Animals

The animal studies used 180 male C57BL/6 mice, weighing between 22 and 25 g and aged 8–10 weeks, sourced from the Animal Experiment Center of Chongqing Medical University. The mice were housed under a 12‐h light/dark cycle at 24°C with 50 ± 1% humidity and had unrestricted access to food and water. The sample sizes for the animal studies were calculated using the sample size calculator from the Chinese University of Hong Kong (http://www.lasec.cuhk.edu.hk/sample‐size‐calculation.html). The mice were randomly divided into six groups as follows: (1) sham, (2) TBI, (3) TBI+oe‐circ NC, (4) TBI+overexpressing circSpna2 (oe‐circSpna2), (5) TBI+sh‐circ NC and (6) TBI+sh‐circSpna2. The sham group served as the baseline control, receiving a sham operation without TBI induction. The TBI group received a TBI without additional treatment, serving as the TBI control. In the TBI+oe‐circ NC group, the mice received TBI, followed by an injection of a lentiviral vector overexpressing a non‐targeting control sequence, allowing for the assessment of vector effects. The TBI+oe‐circSpna2 group received TBI, followed by an injection of a lentiviral vector oe‐circSpna2, which increased circSpna2 levels to evaluate their protective effects against TBI‐induced damage. The TBI+sh‐circ NC group received TBI, followed by an injection of a lentiviral vector containing a non‐targeting control sequence for knockdown, which served as a control for the vector effects. Finally, the TBI+sh‐circSpna2 group received TBI, followed by an injection of a lentiviral vector to knockdown circSpna2, reducing circSpna2 levels to study their role in TBI‐induced damage. The behavioural studies were conducted using 15 mice per treatment group. This ensured adequate statistical power to detect significant differences between groups. The remaining mice were used for the subsequent experiments. The experimenters were blinded to the animal grouping.

### 2.6 Lentivirus injection

A 5% isoflurane solution was used to anaesthetise the mice. After successful anaesthesia, they were positioned on a stereotaxic apparatus. Lentiviral injections were administered at three sites in the left parietal of the cortex, with 3 µL of lentivirus in phosphate‐buffered saline (PBS) delivered at each site: (1) Anterior–posterior (AP) 1.0 mm, Lateral (L) 1.5 mm, Height (H) 1.0 mm; (2) AP 1.0 mm, L 2.5 mm, H 1.0 mm; and (3) AP 1.5 mm, L 2.0 mm, H 1.0 mm (David Kopf Instruments). Fourteen days post‐injection, the left parietal of the cortex, which received the injections, was lesioned to establish the controlled cortical impact (CCI) model.

Lentivirus oe‐circSpna2 and the control (oe‐circ‐NC) were obtained from GeneSeed. The circSpna2 knockdown lentivirus (sh‐circSpna2) and control (sh‐circ‐NC) were obtained from Hanbio. Both lentiviral systems utilised the hSyn (human synapsin‐1) promoter, which drives neuron‐specific gene expression. The hSyn promoter ensures that gene expression is restricted to neurons, preventing expression in non‐neuronal cells such as glial cells. This neuron‐specific expression is critical for accurately studying neuronal processes and molecular mechanisms involved in TBI. The specificity of the hSyn promoter has been validated in numerous studies and is widely used in neurobiological and behavioural research.^33^, ^34^

### 2.7 Controlled cortical impact model

The CCI model was created following established protocols.^24^ In brief, mice were sedated using 5% isoflurane and kept in an environment of 20% oxygen/80% air. TBI group mice were positioned prone, with their heads fixed in a stereotaxic apparatus, followed by a scalp incision and the drilling of a hole in the skull. The specifics of CCI were an impact velocity of 5.0 m/s, a depth of 1.5 mm and a dwell time of 100 ms. After impact, the mice were swiftly removed from the stereotaxic apparatus. Sham group mice underwent the same procedure but without CCI injury. The successful establishment of CCI mice was confirmed by grasping test results for the contralateral limb 2 h post‐TBI.

These mice were used for the following experiments. Mice were included in the contralateral limb grasping test if they underwent a successful CCI procedure, confirmed by observable behavioural and neurological deficits immediately following the procedure, and exhibited normal baseline grasping ability with both forelimbs before the CCI procedure, as determined by pre‐surgery behavioural assessments. Mice were excluded from the experiment if they exhibited any pre‐existing neurological or motor deficits prior to the CCI procedure; did not undergo a complete and successful CCI procedure, as indicated by the absence of expected immediate post‐procedure deficits; or developed severe complications (e.g., infection, excessive bleeding or other health issues) following the CCI procedure.

### 2.8 Whole‐transcriptome resequencing and bioinformatics analysis

The RNA extraction and sequencing methods used were identical to those in our previous study.^24^ Briefly, the extracted RNA was sequenced using a BGI platform (BGI‐Shenzhen). Raw RNA‐sequencing (RNA‐seq) data were processed using the following computational tools: SOAPnuke (v1.5.2) for data filtering,^35^ HISAT2 (v2.0.4) for mapping clean reads,^36^ Bowtie2 (v2.2.5) for aligning clean reads,^37^ and RSEM (v1.2.12) for calculating gene expression levels.^38^ The ‘ggplot’ package in R was used to create a heatmap. DESeq2 (v1.4.5)^39^ was used to analyse differential expression. The circRNAs conforming to a |fold change|>1 and *p* ≤ .05 were deemed differentially expressed genes. CircBase (http://www.circbase.org/) was used to obtain the circRNA sequences and compare circRNA homology between humans and mice. The HDDOCK SERVER (http://hdock.phys.hust.edu.cn/) was used to predict the interaction sites of circRNA and proteins.

### 2.9 Behavioural tests

#### 2.9.1 Open field test

The open field test (OFT) was conducted following the procedures outlined in a previous study.^40^ Mice were positioned in the middle of the open field setup, which is an open Plexiglas container featuring opaque white segments. The field was divided into 25 squares, with the nine central squares designated as the central zone. We measured the duration of the time the mice occupied the central zone in a 5‐min interval.

#### 2.9.2 Tail suspension test

The tail suspension test (TST) was conducted following established protocols.^40^ Each mouse had its tail secured with adhesive tape, and its nose was positioned 25 cm above the tabletop. A 4 cm hollow, rigid and smooth plastic tube encased the tail to prevent tail climbing. The activity of each mouse was monitored for 5 min. The observation metrics included the duration of rest and the time taken to start resting.

#### 2.9.3 Sucrose preference test

The sucrose preference test (SPT) was performed according to established methods.^40^ Prior to the SPT, mice were acclimated to two similar water bottles over a period of 3 days. Each mouse received a 1% sucrose solution and water after 24 h of food and water deprivation. After 12 h, the remaining liquid in the bottles was measured to determine sucrose preference, which was calculated using the formula: [sucrose water intake/(sucrose water intake + plain water intake)] × 100%.

### 2.10 Transmission electron microscopy analysis

For transmission electron microscopy (TEM) analysis, the 1 mm^3^ brain specimens derived from the ipsilesional cortex tissues were prepared according to established methods.^41^ Tissue samples underwent a process of fixation and dehydration and then were sliced into 70 nm‐thick slices. Next, the slices were placed on a grid and stained with lead citrate and uranium acetate. Finally, they were examined under a transmission electron microscope (JEOL JEM‐1400PLUS).

### 2.11 Cell culture and transfection

Mouse hippocampal neuron cells (HT22 cells) were cultured under conditions of 37°C and 5% CO_2_. To overexpress and knockdown Keap1, Nrf2, Atp7b, HA‐ubiquitin, myc‐Nrf2 and Flag‐Keap1, plasmids were sourced from Tsingke Biotechnology Co. To further verify whether circSpna2 expression is affected by inflammatory stimulation, we conducted an additional lipopolysaccharide (LPS) treatment experiment. HT22 cells were treated with LPS (100 ng/mL and 500 ng/mL, S1732‐5mg, Beyotime) for 6, 12 and 24 h. Cells were treated with H_2_O_2_ (600 µM/L) for 6 h, followed by lentivirus or plasmid transfection using LipofectamineTM 2000 (Life Technologies) according to the instructions of the manufacturer, and transfection efficiency was assessed using quantitative real‐time (qRT)‐PCR on day 7 post‐transfection.

293T cells were also cultured under conditions of 37°C and 5% CO_2_. Transfection was initiated when the cells reached 70% confluence. The Nrf2 plasmid and various Atp7b mutant plasmids (Atp7b mut1, Atp7b mut2, Atp7b mut3 and Atp7b mut4) were sourced from Tsingke Biotechnology Co. Each plasmid was individually transfected into separate cell cultures using Lipofectamine 2000.

### 2.12 Quantification of copper

Copper content was quantified as previously described^42^ using a copper assay kit (ab272528, Abcam). Brain tissue samples were homogenised in sodium phosphate buffer (pH 7.5). Different groups of HT22 cells were gathered and homogenised after the addition of distilled water, and reagents were added according to the copper assay kit protocol. After the specified incubation period, absorbance was measured at 359 nm using a microplate reader (Synergy 2, Bio‐Tek Instruments). Protein concentrations were measured using the BCA Protein Assay Kit (Beyotime, catalogue number P0012) to normalise the copper content in the samples. Protein was isolated from the cells or tissue samples utilising a lysis buffer and then centrifuged to remove cell debris. The samples and bovine serum albumin (BSA) standards were placed in a 96‐well plate, to which the working reagent was added. After incubating at 37°C for 30 min, the absorbance was measured at 562 nm using a microplate reader. A BSA standard curve was used to determine the protein concentration in each sample.

### 2.13 Quantitative real‐time PCR

The RNA extraction procedure for brain tissues and HT22 cells was detailed in our previous study.^40^ Briefly, RNA isolation was performed with an RNA extraction kit (Bio‐Tek Instruments). Subsequently, the RNA was configured using reverse transcription components in accordance with the manufacturer's guidelines (RT Master Mix for qPCR, MedChemExpress). Following treatment with RNase R (3 U/mg, 15 min at 37°C), the RT Master Mix from the qPCR kit was utilised for cDNA synthesis. Finally, qRT‐PCR was conducted using SYBR® Green Master Mix (MedChemExpress). The primer sequences can be found in Supporting Information Table 3.

### 2.14 Immunofluorescence and fluorescence in situ hybridisation

Immunofluorescence labelling of the cortex brain slices and HT22 cells was conducted as previously described. Briefly, the brain slices and HT22 cells were rinsed with tris‐buffered saline with tween 20 (TBST) and then treated for 1 h in an antigen retrieval solution. On the first day, the mixed solution of the primary antibody prepared with the dilution solution will be added to the slices and cells for co‐incubation, with the temperature maintained at 4°C. On the second day, the mixed solution will be aspirated and the secondary antibody will be added for co‐incubation. Nuclei were counterstained with 4′,6‐diamidino‐2‐phenylindole (DAPI). The following antibodies we used: Nrf2 (Proteintech, 16396‐1‐AP; dilution 1:200, rabbit), Atp7b (Affinity, AF0410; dilution 1:200, rabbit), Bdnf (Zenbio, 381133; dilution 1:200, rabbit), Keap1 (Affinity, AF5266; dilution 1:200, rabbit), Syn1 (Zenbio, 222757; dilution 1:200, mouse). Normal goat serum was used as the blocking serum. For the co‐localisation study, the circSpna2 probe was designed and manufactured by GeneSeed Biotech. A FISH Kit (RiboBio) was used to detect the probe signals. First, brain slices and HT22 cells were treated with 4% paraformaldehyde and then transferred to a dark room at 37°C overnight for pre‐hybridisation. On the second day, after hybridization with the circSpna2 probe, brain slices or cells were incubated with Keap1 antibody for 1 h, followed by incubation with secondary antibody for 2 h. A confocal microscope (Zeiss LSM800) was used to capture fluorescent images.

### 2.15 Western blotting analysis

Brain tissues or cultured cells were lysed using radioimmunoprecipitation assay buffer (RIPA) buffer containing 1 mM phenylmethylsulfonylfluoride and a protease inhibitor cocktail (catalogue numbers ST506 and P1005, both from Beyotime). The concentration of the extracted protein was measured using a BCA Protein Assay Kit (catalogue number P0012, Beyotime). First, the protein sample underwent sodium dodecyl sulphate‐polyacrylamide gel electrophoresis, followed by electrotransferring of the proteins to a polyvinylidene membrane. Next, the membrane was incubated with the primary antibody overnight at 4°C and with the secondary antibody the next day. An enhanced chemiluminescence (ECL) substrate kit and QuickChemi 5200 Imaging System were employed to detect the protein bands. ImageJ software was used to analyse the relative expression levels of proteins in the bands. A summary of the antibodies, sources and catalogue numbers is provided in Supporting Information Table 4.

### 2.16 Mitochondrial complex I and III activity

The mitochondrial complex activity was evaluated using the CheKine Micro Mitochondrial Complex I and III Assay Kits (KTB1850 and KTB1870, Abbkine) in accordance with the manufacturer's protocols. Brain tissue and HT22 cells were lysed at 4°C, followed by centrifugation at 600 × *g* for 10 min and 11 000 × *g* for 10 min to isolate mitochondria, both at 4°C. The isolated mitochondria were then reconstituted in the assay buffer. Reagents and samples were added to an ultraviolet (UV) plate according to the manufacturer's guidelines. Measurements were taken at the beginning and at 2 min to determine the activity. The absorbance of mitochondrial complex I was measured at 340 nm, and the absorbance of complex III was measured at 550 nm using a Synergy 2 microplate reader (Bio‐Tek Instruments). Enzymatic activity was calculated from changes in absorbance over time and was normalised to the protein content measured using the BCA Protein Assay Kit (Beyotime). Protein was extracted from cells or tissue samples using a lysis buffer, such as RIPA buffer, followed by centrifugation to clear cellular debris. Samples and BSA standards were transferred to a 96‐well plate, and the working reagent was added. Following a 30‐min incubation at 37°C, the absorbance was measured at 562 nm with a microplate reader, and protein concentrations were determined from a BSA standard curve.

### 2.17 Pull‐down assay

The pull‐down assay followed established protocols.^40^ Biotinylated circSpna2 and mutant probes (mut1, mut2, mut3 and mut4) were obtained from GeneSeed. In summary, cells (1 × 10^7^) were exposed to a capture buffer containing 10 µL each of RNase and a protease inhibitor. The supernatant was then collected, and the probes were combined with magnetic beads (MCE). The mixture was centrifuged, separated, and the beads were washed twice with the capture buffer. The beads, now coated with probes, were incubated with the supernatant at 4°C for 1 h with gentle rotation. Next, the beads were removed and washed three times. The RNA–protein complex was treated with sodium dodecyl sulphate buffer and heated to release the proteins. The proteins were precipitated with streptavidin beads (Beyotime Biotechnology) and analyse the proteins using western blotting.

### 2.18 RNA immunoprecipitation assay

For the RNA immunoprecipitation (RIP) assay, full‐length Keap1 and its mutants (Keap1 mut‐BTB, Keap1 mut‐IVR, Keap1 mut‐DGR and Keap1 mut‐CTR) were sub‐cloned into a pCDNA3.1(+) vector with an added HA‐tag at the carboxy‐terminal (Tsingke Biotechnology Co). Subsequently, the HA‐empty vector, HA‐Keap1 and its mutants were introduced into HT22 cells treated with circSpna2 lentivirus for 48 h. Then, the HT22 cells were lysed and incubated with beads coated with anti‐HA‐tagged antibodies (390001, Zenbio). Finally, the co‐precipitated RNAs were extracted and quantified using qRT‐PCR.

### 2.19 Ubiquitin determination

HA‐ubiquitin, myc‐Nrf2 and Flag‐Keap1 were transfected into circSpna2 lentivirus‐treated‐293T cells or 293T cells for 48 h, followed by MG132 (MCE) treatment at 1 µM for an additional 12 h. Subsequently, the 293T cells were lysed. Immunoprecipitation was performed using the anti‐Myc antibody (390003, Zenbio), followed by immunoblotting with an anti‐HA antibody.

### 2.20 Dual‐luciferase reporter system

The method of dual‐luciferase reporter assay was described previously.^40^ The *atp7b* promoter sequence was incorporated into a psiCHECK2 plasmid (HanBio) downstream of the hRluc gene, creating two reporter constructs with putative Nrf2 binding sites. The reporter constructs, along with the Nrf2 plasmid, were simultaneously transfected into 293T cells. Renilla and firefly luciferase activities were assessed with the Dual‐Lumi™ II Luciferase Reporter Gene Assay Kit (Beyotime). The renilla luciferase to firefly luciferase ratio was used to determine the relative luciferase activity.

### 2.21 ChIP‐qPCR

For ChIP‐qPCR, transfected 293T cells were treated with 1% formaldehyde for 10 min at room temperature to stabilise DNA–protein interactions. The fixation reaction was stopped by adding 1.25 M glycine. Cells were lysed in a buffer and sonicated to break the DNA into 200–500 bp fragments. Chromatin was immunoprecipitated overnight at 4°C using an anti‐Nrf2 antibody. Following this, protein A/G magnetic beads were incorporated and incubated for another 2 h. The beads were washed multiple times to remove non‐specific bindings, and the DNA–protein complexes were eluted with a buffer. Reverse cross‐linking was performed overnight at 65°C, and the DNA was subsequently purified using a Qiagen DNA purification kit.

Purified DNA was subjected to qPCR analysis using primers specific to the *atp7b* promoter region. The qPCR reactions utilised SYBR Green Master Mix (Bio‐Rad), specific primers and purified DNA. Amplification was performed on a CFX96 Real‐Time PCR System (Bio‐Rad) with an initial denaturation at 95°C for 10 min, then 40 cycles of 95°C for 15 s and 60°C for 1 min. The results were normalised to the input DNA and assessed using the 2^−ΔΔCt method. Detailed primer sequences for qPCR are provided in Supporting Information Table 3.

### 2.22 Mitochondrial membrane potential

The JC‐1‐Mitochondrial Membrane Potential Assay Kit (M8650, Solarbio) was utilised to assess changes in mitochondrial membrane potential (MMP). Tetraethyl benzimidazole carbocyanine iodide (JC‐1) is a specific cationic dye that forms monomers that fluoresce green at low MMP levels and red or orange fluoresce when it aggregates at high MMP levels. JC‐1 has an absorbance peak at 514 nm, and its fluorescence shifts from green (∼530 nm) to red (∼590 nm) based on the membrane potential. HT22 cells were seeded in 24‐well plates at a density of 25 000 cells per well. After washing with PBS, the cells were mixed with 250 µL of JC‐1 and incubated at 37°C for 20 min in the dark. The cells underwent another PBS wash post‐incubation and were subsequently prepared for analysis. An Olympus DP70 fluorescence microscope was used to capture the fluorescence images.

### 2.23 Sample size determination

The sample sizes for the clinical studies were determined through power analysis. We estimated the expected effect sizes from previous studies and relevant literature. Our goal was to achieve a statistical power of .80 using a significance level of .05. Using these parameters, we calculated the minimum number of participants required using standard statistical software and online tools, including the sample size calculator from the Chinese University of Hong Kong (http://www.lasec.cuhk.edu.hk/sample‐size‐calculation.html). Practical considerations, such as participant availability and feasibility, were also taken into account to ensure adequate sample sizes for reliable and valid results.

### 2.24 Statistical analysis

Data are presented as mean ± SEM. Group averages were evaluated using a two‐tailed *t*‐test, while one‐way analysis of variance (ANOVA) followed by Tukey's post hoc test was used for comparisons between multiple groups. Confocal microscopy image analyses, including background subtraction, noise reduction, quantitative measurements of fluorescence intensity and co‐localisation studies, were carried out using ImageJ (Fiji distribution). GraphPad Prism 9 (v9.4.1) software was used for statistical analysis. A *p* value less than .05 was considered significant.

## 3 RESULTS

### 3.1 CircSpna2 is downregulated in TBI model mice and H_2_O_2_‐treated HT22 cells, and its homologous sequence hsa_circ_0088825 plays an important role in post‐TBI depression

Previous RNA‐seq analysis (PRJNA725662) of sham and TBI mouse brain specimens revealed the significant differential expression of circRNAs, indicating their potential involvement in TBI pathology. We hypothesise that specific circRNAs might play crucial roles in the molecular mechanisms underlying TBI‐induced alterations and could be employed as indicators or therapeutic targets for TBI‐associated conditions, including depression.

**FIGURE 1:**
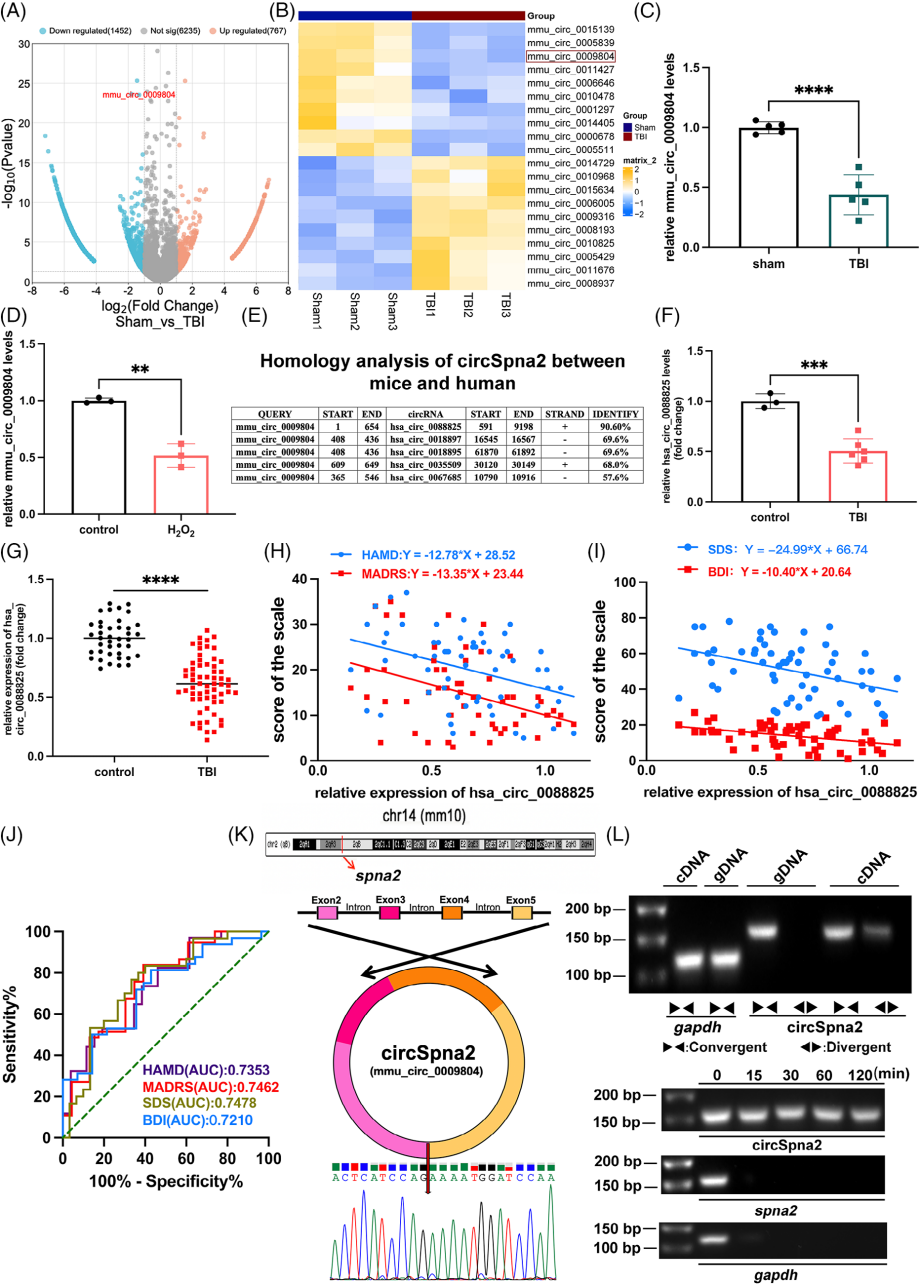
Mmu_circ_0009804 is downregulated in traumatic brain injury (TBI) mice and H_2_O_2_‐treated cells. Hsa_circ_0088825 was elevated in patients with acute TBI and was associated with depressive‐like behaviours after TBI. (A) Volcano plot showing differential RNAs in the TBI group compared with the sham group (fold change ≥1, *p* < .05). (B) Clustered heatmap of differential circular RNAs (circRNAs) screened by the differentially expressed genes (DEG) algorithm showing the top 10 upregulated and downregulated circRNAs (sham group vs. TBI group; fold change ≥ 1, *p* < .05). (C) Relative mmu_circ_0009804 levels were measured by quantitative real‐time‐polymerase chain reaction (qRT‐PCR) in the sham and TBI groups 3 days after TBI (*n* = 5 mice per group); ****p < .0001, two‐tailed *t*‐test. (D) Relative mmu_circ_0009804 levels in H_2_O_2_‐treated HT22 cells and the control group (*n* = 3 replicates); **p < .01, two‐tailed *t*‐test. (E) Homology analysis between mice mmu_circ_0009804 and human circRNAs. (F) Relative expression levels of hsa_circ_0088825 in brain tissue samples from TBI patients (*n* = 6 TBI patients, *n* = 3 healthy controls); ***p < .001, two‐tailed *t*‐test. (G) Relative expression levels of hsa_circ_0088825 in the plasma of patients with acute TBI, which was highly homologous to mmu_circ_0009804, and downregulated after TBI (*n* = 60 TBI patients, *n* = 40 healthy controls); ****p < .0001, two‐tailed *t*‐test. (H) Depressive‐like behaviours were analysed using the Hamilton Depression Scale (HAMD) and Montgomery–Åsberg Depression Rating Scale (MADRS) 3 months after TBI. Both scores had a negative linear relationship with hsa_circ_0088825 expression (*n* = 60 TBI patients, *n* = 40 healthy controls). HAMD: *Y* = −12.78 × *X* + 28.52, *p* = .0025; MADRS: *Y* = −13.35 × *X* + 23.44, *p* = .0020. (I) Depressive‐like behaviours were analysed using the self‐rating anxiety scale (SDS) and Beck Depression Inventory (BDI) 3 months after TBI. Both scores had a negative linear relationship with hsa_circ_0088825 expression (*n* = 60 TBI patients, *n* = 40 healthy controls). SDS: *Y* = −.24.99 × *X* + 66.74, *p* = .0017; BDI: *Y* = −10.40 × *X* + 20.64, *p* = .0014. (J) Receiver operating characteristic (ROC) curve for the diagnostic value of plasma hsa_circ_0058195 for anxiety after TBI. HAMD: area under the curve (AUC) = .7353, *p* = .0019; MADRS: AUC = .7462, *p* = .0014; SDS: AUC = .7478, *p* = .0010; BDI: AUC = .7210, *p* = .0033. (K) Schematic representation of the circularisation of Spna2 2–5 exons to form mmu_circ_0009804 (circSpna2). Sanger sequencing results of the spliced junction resulting from the divergent primers. (L) The presence of circSpna2 was validated in HT22 cells by PCR and agarose gel electrophoresis (AGE). Divergent primers amplified circSpna2 from cDNA but not from gDNA. CircSpna2, linear Spna2 and GAPDH levels were detected by PCR and AGE treated with RNase R for 0–120 min. All data are presented as the mean ± SEM.

In our previous study, the RNA‐seq analysis (PRJNA725662) of sham and TBI mouse brain specimens^24^ revealed 1452 upregulated and 767 downregulated circRNAs (both with | fold change |>1, *p* < .05; Figure 1A). The circRNAs were differentially expressed post‐TBI, and we screened ten upregulated and ten downregulated circRNAs (Figure 1B). In this study, we performed qRT‐PCR analysis of the 20 circRNAs in vivo and in vitro. We observed a marked downregulation of circSpna2 in the brain tissues of TBI mice (*p* < .0001, Figure 1C) and HT22 cells treated with H_2_O_2_ (*p* < .01, Figure 1D). In addition, we conducted experiments to examine whether circSpna2 expression is altered by LPS‐induced inflammation. HT22 cells were treated with two concentrations of LPS (100 ng/mL and 500 ng/mL) for 6, 12 and 24 h. As shown in Supporting Information Figure 1A,B, circSpna2 expression remained unchanged across all time points and LPS concentrations when compared to the control group, indicating that circSpna2 is not significantly regulated by inflammatory responses. Subsequently, we confirmed the homology between mmu_circ_0009804 (circSpna2) and human circRNAs. We found that the host genes for mmu_circ_0009804 and hsa_circ_0088825 were homologous mouse *spna2* and human *SPNA2* genes (Figure 1E), and mmu_circ_0009804 had a high sequence similarity (90.6%) to hsa_circ_0088825. The expression levels of hsa_circ_0088825 were significantly reduced in TBI patients compared to healthy controls. Specifically, the expression in brain tissue samples of TBI patients was significantly lower (*p* < .001, Figure 1F). Additionally, the expression levels in plasma samples from TBI patients were significantly lower compared to healthy controls (*p* < .0001, Figure 1G). Two other evaluations (HAMD and MADRS) and two self‐evaluations (SDS and BDI) were used to explore the link between hsa_circ_0088825 and mood disorders. Hsa_circ_0088825 was negatively correlated with HAMD, MADRS, SDS and BDI scores (Figure 1H,I). The analysis of the receiver operating characteristic (ROC) curve indicated that hsa_circ_0088825 had good sensitivity for diagnosing post‐TBI depressive‐like behaviours (Figure 1J).

We stratified the analysis by gender to address the potential impact of gender differences on circSpna2 expression variability and ensure comprehensive sampling and analysis. In both male and female TBI patients, the expression of hsa_circ_0088825 was markedly lower compared to healthy controls (Supporting Information Figure 1C,D). In both males and females, hsa_circ_0088825 levels were inversely associated with HAMD, MADRS, SDS and BDI scores (Supporting Information Figure 1E–H). ROC curve analysis indicated that hsa_circ_0088825 demonstrated good sensitivity for diagnosing post‐TBI depressive‐like behaviours in both males and females (Supporting Information Figure 1I,J).

In the mouse model, we conducted gender‐stratified analyses to evaluate circSpna2 expression. The findings indicated that mmu_circ_0009804 levels in TBI mice were significantly reduced compared to the sham group in both sexes on days 1, 3, 7, 15 and 30 post‐TBI (Supporting Information Figure 1K,L). The preliminary data indicated similar expression trends across genders, and we plan to include more detailed analyses in future studies.

Moreover, we expanded our analysis to further investigate the temporal dynamics of circSpna2 expression in specific brain regions. Our time‐course analysis revealed a persistent and significant downregulation of circSpna2 in the ipsilateral cortex and hippocampus at multiple time points post‐TBI, including days 1, 3, 7, 15 and 30 (Supporting Information Figure 2A,B). In contrast, there were no significant changes in circSpna2 expression in the contralateral cortex or hippocampus throughout the same time points (Supporting Information Figure 2C,D). These findings underscore that circSpna2 downregulation is a localised, sustained response in the injury‐affected regions.

CircSpna2 was spliced 654 bp from exons 2–5 of Spna2 (Figure 1K). CircSpna2 was not detectable by PCR analysis using divergent genomic DNA (gDNA) primers but was amplifiable from cDNA. In contrast, linear Spna2 was detectable in both cDNA and gDNA using convergent primers. Because circRNA is cyclised, linear Spna2 showed significantly lower stability compared to circSpna2 when subjected to RNase R treatment (Figure 1L).

These findings suggest that circSpna2, particularly hsa_circ_0088825, is significantly downregulated in TBI and associated with depressive symptoms. Its homology and correlation with mood disorder scale scores highlight its potential as a biomarker for post‐TBI depression. The stability and specific expression patterns of circSpna2 further support its relevance in TBI pathology.

### 3.2 Overexpression of circSpna2 alleviates depressive‐like behaviours in TBI mice

Based on the observation that circSpna2 is downregulated in TBI and potentially implicated in mood disorders, we hypothesised that the overexpression of circSpna2 could mitigate depressive‐like behaviours in a TBI mouse model.

**FIGURE 2:**
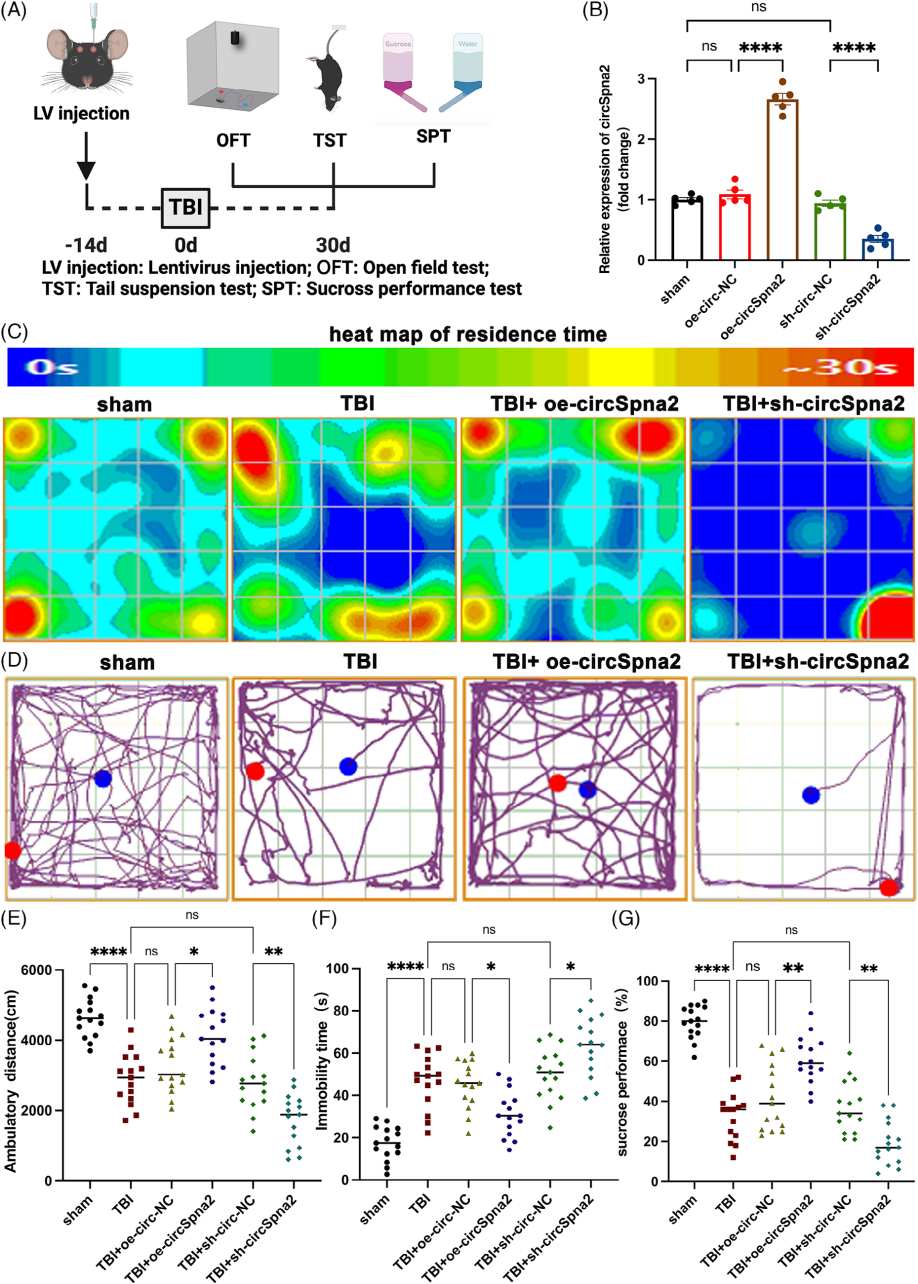
CircSpna2 relieves depressive‐like behaviours in mice after traumatic brain injury (TBI). (A) Illustration of the experimental procedure. (B) The relative expression level of circSpna2 after the injection of lentivirus overexpressing or inhibiting circSpna2 into the left parietal lobe of mice for 14 days (*n* = 5 mice per group); overexpressing circSpna2 (oe‐circSpna2) versus oe‐circ‐NC, ****p < .0001, sh‐circSpna2 versus sh‐circ‐NC, ****p < .0001 by one‐way repeated measures analysis of variance (ANOVA), followed by Tukey's multiple comparisons test. *Note*: The TBI group data were not included in this panel as the primary objective was to validate the efficiency of lentivirus‐mediated circSpna2 expression, rather than comparing TBI versus non‐TBI conditions. (C–E) Depressive‐like behaviours were measured using the open field test (OFT) 30 days after TBI. The heatmap and representative OFT trails (*n* = 15 mice per group). (C) Heatmap of residence time. (D) Moving trails. (E) Ambulatory distance: *F* = 29.16, *p* < .0001; TBI versus sham, ****p < .0001; TBI+oe‐circSpna2 versus TBI+oe‐circ‐NC, *p < .05; TBI+sh‐circSpna2 versus TBI+sh‐circ‐NC, **p < .01 by one‐way repeated measures ANOVA, followed by Tukey's multiple comparisons test. (F) Depressive‐like behaviours were measured by the tail suspension test (TST) 30 days after TBI (*n* = 15 per group). *F* = 28.17, *p* < .0001. TBI versus sham, ****p < .0001; TBI+oe‐circSpna2 versus TBI+oe‐circ‐NC, *p < .05; TBI+sh‐circSpna2 versus TBI+sh‐circ‐NC, *p < .05 by one‐way repeated measures ANOVA, followed by Tukey's multiple comparisons test. (G) Depressive‐like behaviours were measured by the sucrose preference test (SPT) 30 days after TBI (*n* = 15 per group). *F* = 46.33, *p* < .0001. TBI versus sham, ****p < .0001; TBI+oe‐circSpna2 versus TBI+oe‐circ‐NC, **p < .01; TBI+sh‐circSpna2 versus TBI+sh‐circ‐NC, ** *p* < .01 by one‐way repeated measures ANOVA, followed by Tukey's multiple comparisons test. One‐way ANOVA followed by Tukey's multiple comparisons test. ns, no significant difference. All data are presented as the mean ± SEM.

To explore whether circSpna2 alleviated mood disorders in TBI model mice, we injected lentivirus to overexpress or knockdown circSpan2 in the left parietal lobe cortex of the mice 14 days before TBI. At 30 days post‐TBI, depressive‐like behaviours were assessed by behavioural tests (Figure 2A). The expression of circSpna2 after lentivirus injection for 14 days was determined by qRT‐PCR, as shown in Figure 2B. In addition, Supporting Information Figure 2E showed the dynamic expression changes of circSpna2 in different treatment groups (sham, TBI, TBI+oe‐circ‐NC, TBI+oe‐circSpna2, TBI+sh‐circ‐NC and TBI+sh‐circSpna2) from day 0 to day 30 post‐TBI. In the TBI+oe‐circSpna2 group, circSpna2 expression was significantly upregulated compared to the control groups (TBI+oe‐circ‐NC), whereas in the TBI+sh‐circSpna2 group, circSpna2 expression was markedly downregulated compared to the control groups (TBI+sh‐circ‐NC). These results further confirmed the effective modulation of circSpna2 expression in the TBI model across multiple time points. The heatmap of the OFT indicated that circSpna2 overexpression markedly extended the central exploration time in TBI mice, while its knockdown countered this effect (Figure 2C). The track map for the OFT showed that overexpression of circSpna2 significantly increased the ambulatory distance of TBI mice, whereas inhibition of circSpna2 significantly reduced ambulatory distance of TBI mice (Figure 2D,E). The TST results showed that overexpression of circSpna2 reduced immobility time (*p* < .05, Figure 2F), indicating a reduction in TBI‐induced depressive‐like behaviours. Similarly, the SPT results showed that overexpression of circSpna2 increased sucrose preference (*p* < .01, Figure 2E), further demonstrating its effect in reducing TBI‐induced depressive‐like behaviours.

To further investigate the effect of circSpna2 on depressive‐like behaviours in wild‐type mice, we conducted additional behavioural tests, including the OFT, TST and SPT in sham‐operated mice injected with sh‐circSpna2. The results showed that sh‐circSpna2 treatment did not induce significant changes in locomotor activity (Supporting Information Figure 2F), immobility time in the TST (Supporting Information Figure 2G) or sucrose preference in the SPT (Supporting Information Figure 2H). These results indicate that circSpna2 knockdown in wild‐type mice did not induce depressive‐like behaviours under normal conditions.

These findings indicate that the overexpression of circSpna2 can mitigate depressive‐like behaviours in TBI mice, supporting its potential therapeutic role in TBI‐induced mood disorders.

### 3.3 Overexpression of circSpna2 alleviates cuproptosis and synapse dysfunction in TBI mice

Building on our previous findings that the overexpression of circSpna2 alleviated depression‐like behaviours in TBI mice, the role of circSpna2 in this process was investigated, particularly focusing on copper levels, mitochondrial function and related protein expression. Cu is an essential trace element, and its imbalance can lead to various pathophysiological changes. Numerous studies have confirmed that elevated Cu levels can induce depression‐like behaviours in various neurological disease models.^43^ For example, exposure to low doses of copper intensified depression‐like behaviour in ApoE4 transgenic mice.^44^ Copper was shown to trigger oxidative stress and neuronal apoptosis in the hippocampus, influencing the CREB/Bdnf and Nrf2/HO‐1/NQO1 signalling pathways.^45^

**FIGURE 3:**
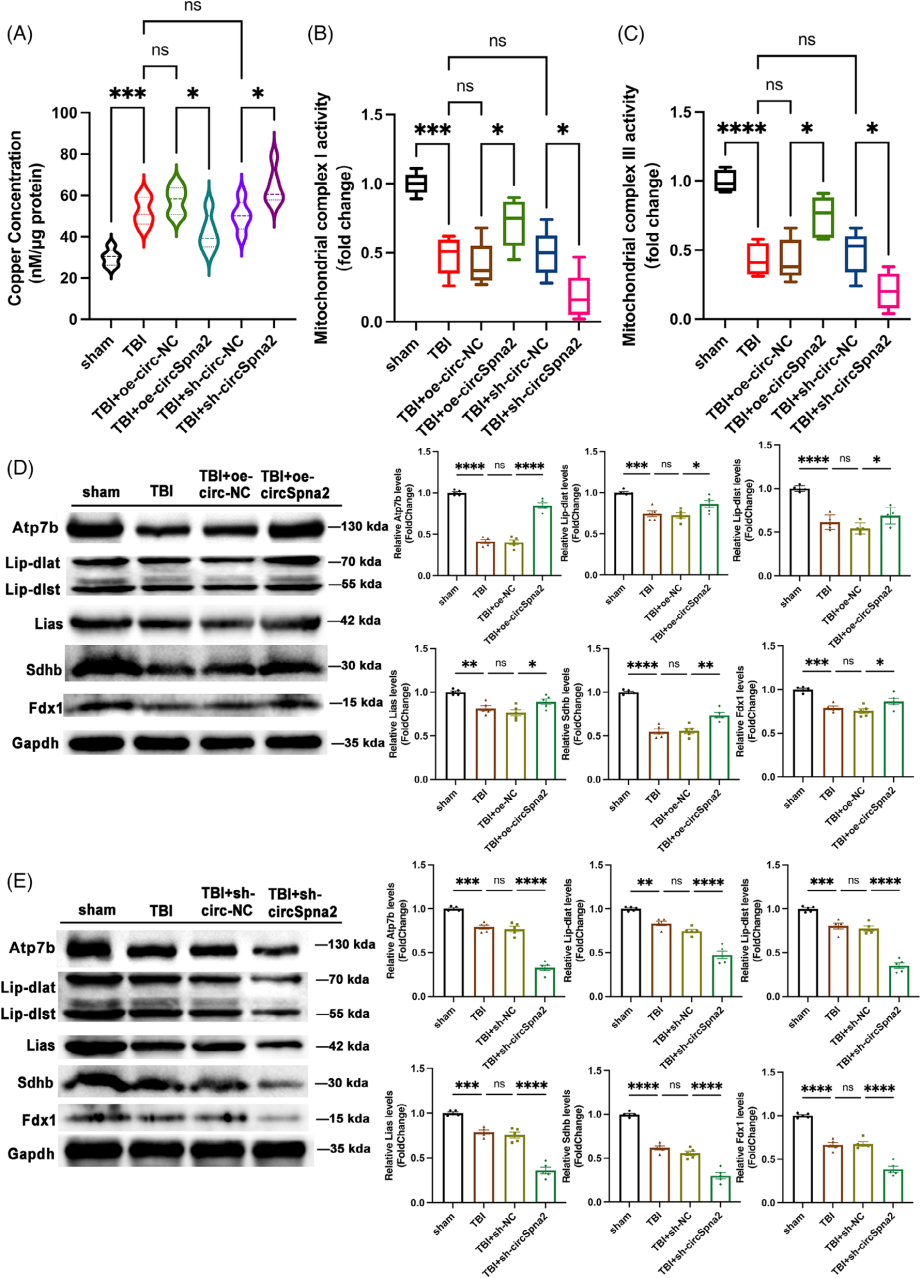
CircSpna2 reduces cuproptosis in traumatic brain injury (TBI) mice. (A) Copper ion concentrations in circSpna2‐overexpressing (oe‐circSpna2) or knockdown (sh‐circSpna2) mice post‐TBI (*n* = 5 per group). (B) Mitochondrial complex I levels in circSpna2‐overexpressing or knockdown mice 3 days post‐TBI (*n* = 5 per group). (C) Mitochondrial complex III levels in circSpna2‐overexpressing or knockdown mice 3 days post‐TBI (*n* = 5 per group). (D and E) Expression of Atp7b, Lip‐dlat, Lip‐dlst, Lias, Sdhb and Fdx1 in circSpna2‐overexpressing (D) or knockdown (E) mice 3 days post‐TBI determined by western blotting (*n* = 5 per group). Detailed *p* values for each comparison are provided. Data are presented as the mean ± SEM. One‐way analysis of variance (ANOVA) was used, followed by Tukey's multiple comparisons test. *p < .05, **p < .01, ***p < .001, ****p < .0001, ns, not significant.

The role of copper in inducing depressive‐like behaviours is multifaceted. Elevated copper levels can promote the formation of ROS, leading to oxidative stress and neuronal damage.^16^ This oxidative stress can impair mitochondrial function, the energy powerhouses of cells, disrupting energy metabolism and leading to cellular dysfunction.^46^ Oxidative stress can also influence the levels and activity of neurotransmitters and neurotrophic factors, such as brain‐derived neurotrophic factor (Bdnf), which are crucial for maintaining synaptic plasticity and neuronal health.^45^ The dysregulation of these factors is closely associated with mood disorders, including depression. Studies have shown that copper exposure can reduce Bdnf expression, thereby impairing synaptic function and contributing to depressive‐like behaviours.^47^ Building on these insights, our analysis revealed that cuproptosis, a newly identified type of programmed cell death, was triggered by copper imbalances and significantly impacted mitochondrial function, especially the activity of mitochondrial complexes I and III.^16^ Our study demonstrated that the overexpression of circSpna2 decreased copper ion levels and significantly increased mitochondrial complex I and III activities, whereas the knockdown of circSpna2 aggravated these changes (Figure 3A–C). Next, we aimed to detect the expression of several cuproptosis‐related proteins, including Atp7b, lipoacylation‐associated proteins (Lip‐dlat and Lip‐dlst) and Fe–S cluster proteins (Lias, Sdhb and Fdx1). These proteins play critical roles in cellular processes affected by cuproptosis. Atp7b is involved in copper transport and homeostasis, Lip‐dlat and Lip‐dlst are linked to the lipoacylation process necessary for mitochondrial function, and Lias, Sdhb and Fdx1 are components of the mitochondrial Fe–S cluster machinery crucial for various enzymatic activities. Notably, the overexpression of circSpna2 alleviated the loss of these proteins, whereas knockdown exacerbated their reduction (Figure 3D,E).

**FIGURE 4:**
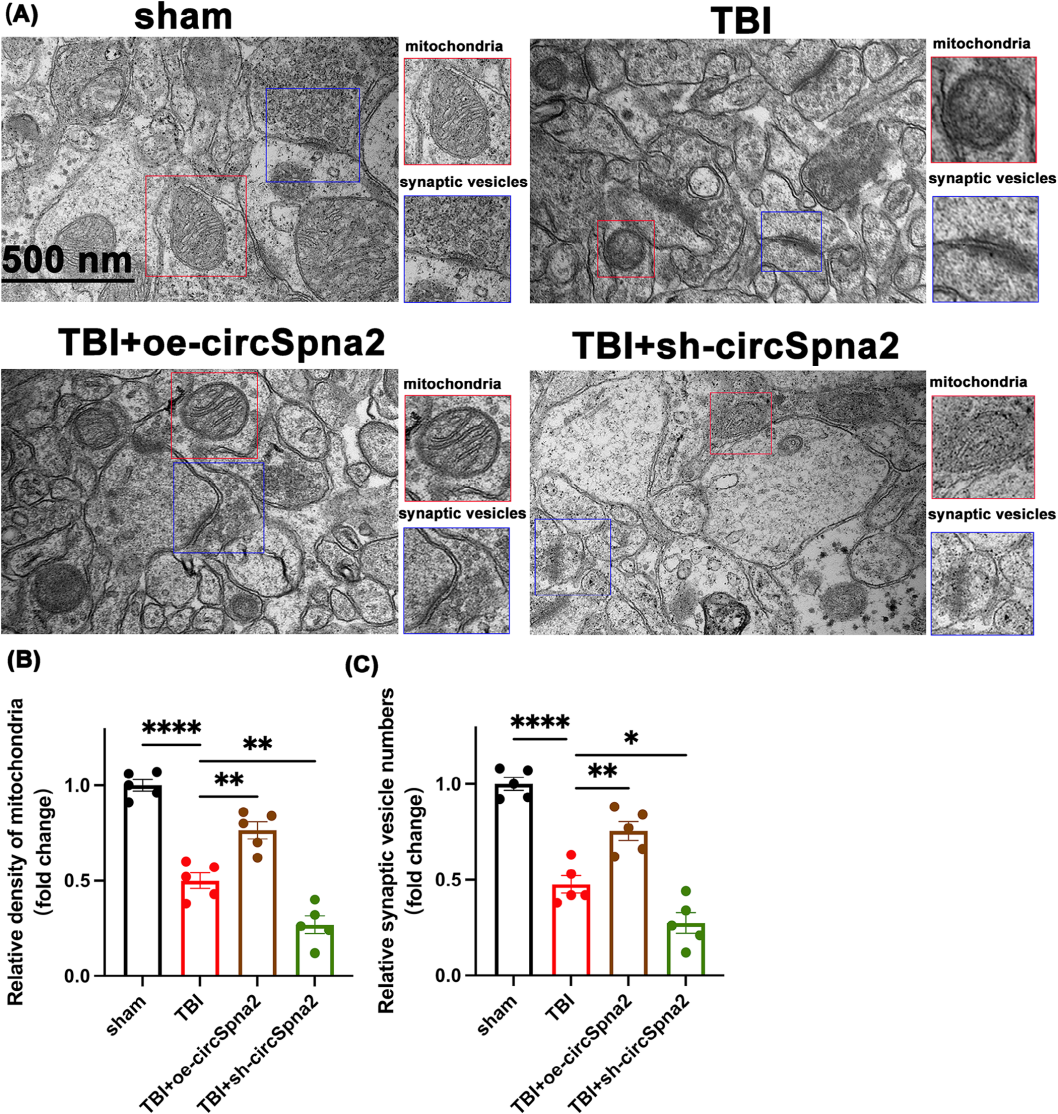
CircSpna2 modulates mitochondrial and synaptic ultrastructure post‐traumatic brain injury (TBI). (A) Ultrastructural changes in mitochondria and synaptic vesicles post‐circSpna2 modulation 3 days post‐TBI, observed by transmission electron microscopy (TEM). The blue‐boxed regions indicate mitochondria and the red‐boxed regions indicate synaptic vesicle numbers. *Note*: The selection of groups in panel A focused on illustrating the direct effects of circSpna2 overexpression and knockdown on mitochondrial and synaptic structures under TBI conditions. Including the negative control groups (NC groups) was not deemed necessary for this panel, as they would not have provided additional insights into the specific ultrastructural changes being investigated. This selective inclusion aimed to provide a clearer understanding of the morphological impacts of circSpna2 modulation in the context of TBI. (B and C) In the TBI group, cristae density was notably decreased, and the number of synaptic vesicles was significantly reduced compared to the control group. These adverse changes were ameliorated in the TBI+overexpressing circSpna2 (oe‐circSpna2) group and exacerbated in the TBI+sh‐circSpna2 group. Detailed *p* values for each comparison are provided. Data are presented as the mean ± SEM. One‐way analysis of variance (ANOVA) was used, followed by Tukey's multiple comparisons test. *p < .05, **p < .01, ***p < .001, ****p < .0001, ns, not significant.

Cuproptosis impacts not only the molecular but also the ultrastructural level, causing damage to mitochondria and synaptic junctions. TEM experiments were conducted to investigate these effects by providing high‐resolution images revealing detailed alterations in mitochondrial density, cristae structure and synaptic junctions. These findings are critical for understanding the cellular and molecular mechanisms underlying neuroprotection by circSpna2. Investigating ultrastructural changes using TEM is essential for understanding how cuproptosis‐induced mitochondrial damage can impair cellular function and contribute to neurodegenerative processes. Synaptic loss and dysfunction are hallmark features of neurodegenerative diseases and depression. By examining synaptic changes, we can understand how circSpna2 preserves neuronal communication and plasticity. TEM analysis complements our molecular findings by providing visual evidence of the structural integrity of mitochondria and synapses, thereby validating the protective effects of circSpna2 at a cellular level. TEM analysis revealed that circSpna2 overexpression could reverse ultrastructural changes, such as reduced mitochondrial density caused by mitochondrial swelling and altered synaptic vesicle numbers, whereas knockdown worsened the damage. Specifically, the TBI group exhibited markedly swollen mitochondria, the loss of cristae structure and a reduction in the number of synaptic vesicles. These changes were improved significantly in the TBI+oe‐circSpna2 group and worsened in the TBI+sh‐circSpna2 group. Cristae density and synaptic vesicle numbers were measured using ImageJ software to quantitatively assess these differences. Statistical analysis revealed a significant decrease in cristae density (*p* < .0001) and a significant reduction in synaptic vesicle numbers (*p* < .0001) in the TBI group compared to the control group. These adverse changes were significantly ameliorated in the TBI+oe‐circSpna2 group (*p* < .05) and exacerbated in the TBI+sh‐circSpna2 group (*p* < .05; Figure 4A–C). The neurotrophic hypothesis of depression posits that Bdnf can enhance synaptic plasticity to mitigate depression.^48^ Copper ions have been shown to reduce Bdnf expression. Nrf2/Keap1, a critical regulator of copper ion‐induced oxidative stress, also plays a significant role in regulating cuproptosis.

**FIGURE 5:**
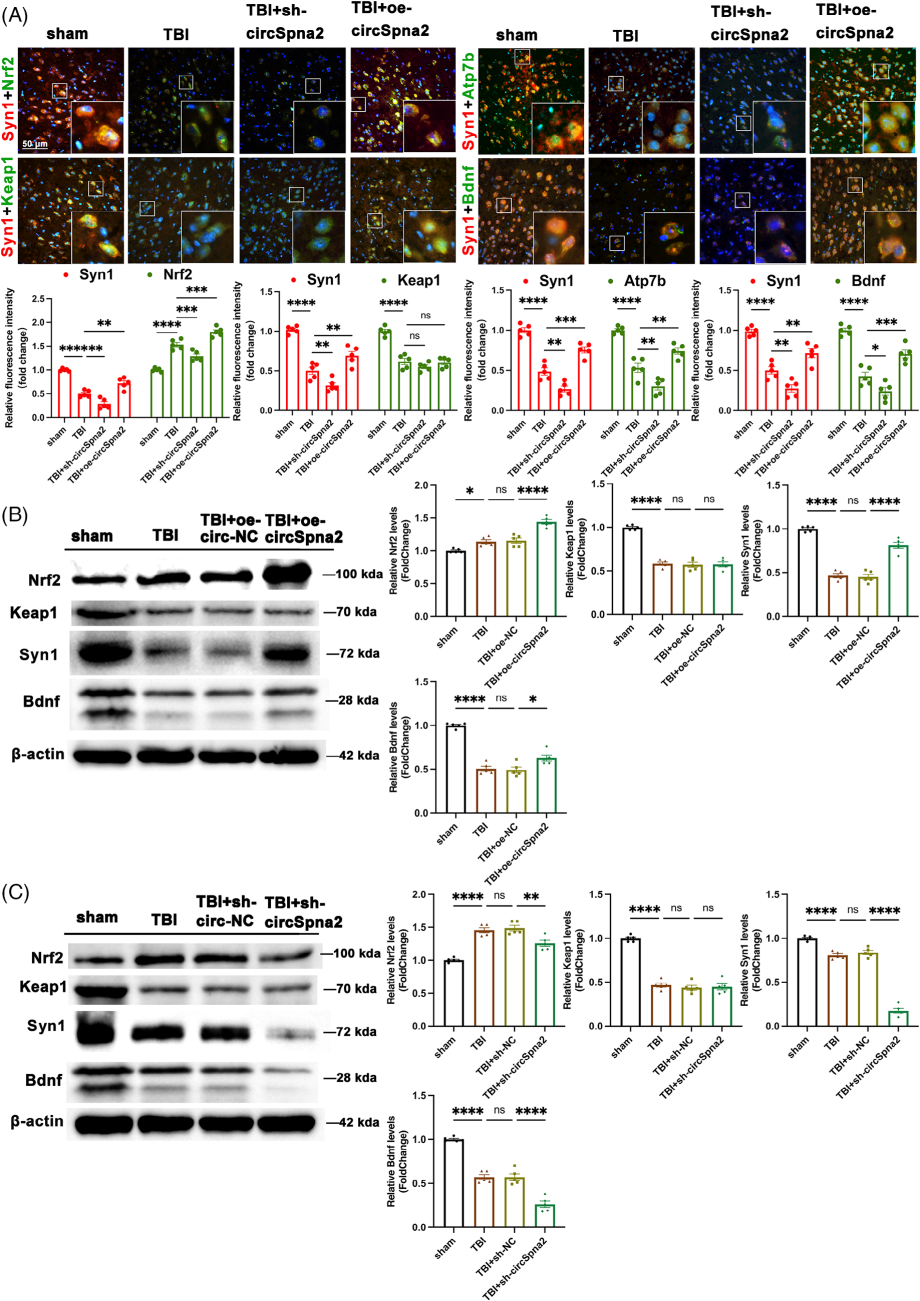
CircSpna2 regulates Nrf2‐Keap1 signalling and synaptic protein interactions in traumatic brain injury (TBI) mice. (A) The co‐localisation of Syn1 with Nrf2, Keap1, Bdnf or Atp7b in sham, TBI, TBI+overexpressing circSpna2 (oe‐circSpna2) and TBI+sh‐circSpna2 groups detected by double immunofluorescence. *Note*: The selection of groups in panel A was specifically focused on highlighting the key interactions of Syn1 with various proteins (Nrf2, Keap1, Bdnf and Atp7b) in the context of TBI and circSpna2 manipulation. Including the negative control groups (NC groups) was not deemed necessary for this panel, as it would not have added additional insights into the specific interactions being investigated. This selective inclusion was intended to provide a clearer understanding of the molecular mechanisms under study. (B and C) The expression of Nrf2, Keap1, Bdnf and Syn1 in circSpna2‐overexpressing (B) or knockdown (C) mice 3 days post‐TBI determined by western blotting (*n* = 5 per group). Detailed *p* values for each comparison are provided. Data are presented as the mean ± SEM. One‐way analysis of variance (ANOVA) was used, followed by Tukey's multiple comparisons test. *p < .05, **p < .01, ***p < .001, ****p < .0001, ns, not significant.

Therefore, we hypothesised that circSpna2 might regulate Nrf2/Keap1 to mitigate Bdnf loss induced by cuproptosis, thus alleviating synapse dysfunction. The detection of related proteins (Bdnf, Syn1, Nrf2 and Keap1) showed that the overexpression of circSpna2 reduced the loss of Syn1 and Bdnf in TBI mice and increased Nrf2 levels, but did not affect Keap1 expression. Conversely, circSpna2 knockdown aggravated the loss of Syn1 and Bdnf and decreased Nrf2 without altering Keap1 expression (Figure 5A–C).

These findings suggest that overexpression of circSpna2 can improve cuproptosis and synapse dysfunction in TBI mice, highlighting its potential therapeutic role in TBI‐induced mood disorders.

### 3.4 CircSpna2 targets the DGR domain of Keap1 to alleviate Nrf2 ubiquitination

Continuing from the findings that the overexpression of circSpna2 mitigated cuproptosis and synapse dysfunction by interacting with the Keap1‐Nrf2 pathway, we hypothesised that circSpna2 might directly interact with the key regulatory proteins involved in oxidative stress and synaptic function. Specifically, we proposed that circSpna2 can play an important role in cellular responses to oxidative stress by modulating the Keap1‐Nrf2 signalling pathway and has significant effects on neuroprotection and depression.

**FIGURE 6:**
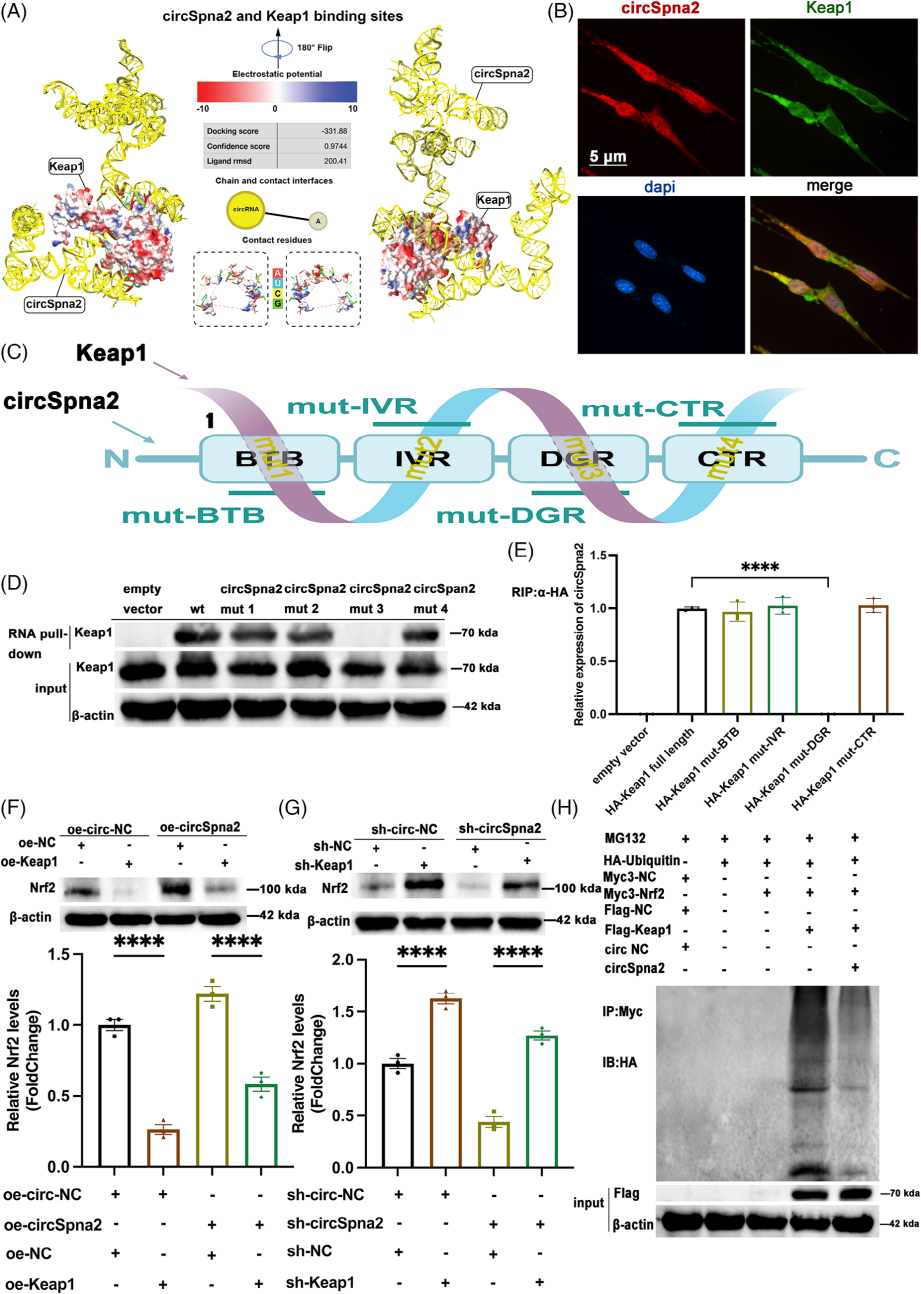
CircSpna2 acts as a Keap1molecular sponge to regulate Nrf2. (A) Circular RNA (circRNA)–protein docking: circSpna2 and Keap1 binding sites. Docking score = −331.88, confidence score = .9744 and ligand rmsd = 200.41. (B) Fluorescence in situ hybridisation (FISH) probes and immunofluorescence results indicated that circSpna2 and Keap1 were enriched in the HT22 cells. (C) Illustration of the predicted circSpna2 and Keap1 binding sites. CircSpna2: mut1 (binding BTB), mut2 (binding IVR), mut3 (binding DGR) and mut4 (binding CTR); Keap1: mut‐BTB, mut‐IVR, mut‐DGR and mut‐CTR. (D) Cytoplasm extracts from HT22 cells incubated with biotinylated empty vector, biotinylated circSpna2, biotinylated circSpna2 mut1, biotinylated circSpna2 mut2, biotinylated circSpna2 mut3 and biotinylated circSpna2 mut4. The proteins were precipitated with streptavidin beads and subjected to immunoblotting (IB) analysis with an anti‐Keap1 antibody. (E) RNA immunoprecipitation (RIP) assay of the interaction between Keap1, Keap1 mut‐BTB, Keap1 mut‐IVR, Keap1 mut‐DGR and Keap1 mut‐CTR with circSpna2 in HT22 cells. CircSpna2 was immunoprecipitated using an anti‐HA antibody, and the relative levels were measured by quantitative real‐time‐polymerase chain reaction (qRT‐PCR; *n* = 3). (F) Expression of Nrf2 after co‐transfection with oe‐circ‐NC+oe‐NC, oe‐circ‐NC+oe‐Nrf2, overexpressing circSpna2 (oe‐circSpna2) + oe‐NC or oe‐circSpna2+oe‐Keap1 (*n* = 3 replicates). (G) The expression of Nrf2 after co‐transfection with sh‐circ‐NC+sh‐NC, sh‐circ‐NC+sh‐Nrf2, sh‐circSpna2+sh‐NC or sh‐circSpna2+oe‐Keap1 (*n* = 3 replicates). (H) Effect of circSpna2 on Keap1‐mediated Nrf2 ubiquitination. 293T cells co‐transfected with the indicated plasmids were treated with MG132 (1 µM) for 12 h and subjected to immunoprecipitation (IP) with an anti‐Myc antibody followed by IB with an anti‐HA antibody. Whole‐cell expression (input) of proteins was detected by IB using anti‐FLAG or anti‐β‐actin antibodies. *Note*: In this figure, the selection of experimental groups was focused on illustrating the key interactions between circSpna2 and Keap1 mutants to demonstrate the specific mechanistic pathways involved. Including all treatment groups could have diluted the clarity of the specific interactions and effects we aimed to highlight. This selective inclusion was intended to provide a clearer understanding of the molecular mechanisms under investigation. Detailed *p* values for each comparison are provided. Data are presented as the mean ± SEM. One‐way analysis of variance (ANOVA) was used, followed by Tukey's multiple comparisons test. *p < .05, **p < .01, ***p < .001, ****p < .0001, ns, not significant.

Molecular docking was performed to determine the underlying mechanism through which circSpna2 regulates cuproptosis and synapse dysfunction. We found that circSpna2 could dock with Keap1 protein (Figure 6A). The co‐localisation staining results showed that circSpna2 and Keap1 were co‐localised in HT22 cells (Figure 6B and Supporting Information Figure 3A). Co‐localisation studies in HT22 cells, as well as brain slices from both control and TBI mice, were performed to explore how circSpna2 associates with Keap1. The co‐localisation staining results showed that circSpna2 co‐localised with Keap1 in HT22 cells (Figure 6B). The co‐localisation staining results in HT22 cells were quantified and shown as grey value intensity profiles in Supporting Information Figure 3A. Co‐localisation staining for circSpna2 and Keap1 in brain slices from both control and TBI mice were also conducted (Supporting Information Figure 3B–D). Additionally, we investigated whether circSpna2 affected the transcription of Keap1. The overexpression of circSpna2 in HT22 cells did not alter Keap1 mRNA levels (Supporting Information Figure 3E), confirming that circSpna2 regulated Nrf2 activity without affecting Keap1 transcription. qRT‐PCR, performed to further study whether the knockdown of circSpna2 affected the expression of its host mRNA Spna2, showed that circSpna2 knockdown did not alter Spna2 mRNA levels in TBI mouse brain tissues (Supporting Information Figure 3F) or HT22 cells (Supporting Information Figure 3G), indicating that the function of circSpna2 was independent of its host mRNA. The off‐target effects often observed in RNAi experiments were ruled out by using three independent shRNA sequences targeting circSpna2. The result showed that sh‐circSpna2_1 demonstrated better knockdown efficiency (Supporting Information Figure 3H).

An image of circSpna2 combined with Keap1 is shown in Figure 6C. The domains of Keap1 included NTR, BTB, IVR, DGR and CTR.^45^ Further exploring the molecular docking results, circSpna2 was found to bind with BTB, IVR, DGR and CTR. We constructed Keap1 fragments with point mutations at the amino acid binding sites of the corresponding domains (mut BTB, mut IVR, mut DGR and mut CTR) to identify the Keap1 domains responsible for binding with circSpna2. The sequences are shown in Supporting Information Table 5. We also generated fragments with point mutations at the nucleotide‐binding sites of circSpna2 (mut1, mut2, mut3 and mut4) to determine the regions responsible for interacting with Keap1, as shown in Supporting Information Table 6. The RNA pull‐down and immunoblotting results showed that the binding sites of circSpna2's interaction with DGR were essential (Figure 6D). The expression of HA‐tagged Keap1 and mutants was validated by western blot (Supporting Information Figure 3I). And RIP assays were performed to confirm the interaction between Keap1 and circSpna2, and the result showed that Keap1 could not bind to circSpna2 when the amino acid binding sites of the DGR domain were mutated (Figure 6E). The DGR domain is the binding site of Keap1 protein with Nrf2 protein,^45^ and circSpna2 may reduce the Keap1‐mediated ubiquitination of Nrf2 by competitively binding the DGR domain with Nrf2.

We transfected Keap1 plasmid or sh‐Keap1 plasmid with oe‐circSpna2 or sh‐circSpna2 lentivirus in HT22 cells to investigate whether circSpna2 regulated the influence of Keap1 on Nrf2 expression. The results showed that the overexpression of circspna2 improved the expression of Nrf2 induced by the Keap1 plasmid (*p* < .0001, Figure 6F), whereas circspna2 knockdown reversed elevations in Nrf2 expression caused by the sh‐Keap1 plasmid (*p* < .0001, Figure 6G). As shown in Figure 6H, we also co‐transfected plasmids in 293T cells with HA‐ubiquitin, Myc3‐Nrf2 and Flag‐Keap1 and added MG132 (5 µM) for 24 h to stimulate Nrf2 ubiquitination via Keap1. The results indicated that this ubiquitination process was significantly reversed by circSpna2 transfection.

These findings elucidate a novel mechanism through which circSpna2 regulates Keap1‐Nrf2 interactions to mitigate cuproptosis and synaptic dysfunction. By targeting the DGR domain of Keap1, circSpna2 effectively reduced the Keap1‐mediated ubiquitination of Nrf2, thereby enhancing the neuroprotective effects of Nrf2.

### 3.5 Nrf2 binds to the *atp7b* promoter

Our previous findings demonstrated that circSpna2 alleviated depression‐like behaviours and mitigated copper‐induced cuproptosis by interacting with the Keap1‐Nrf2 pathway. This led us to hypothesise that Nrf2 might further regulate cuproptosis by directly interacting with key genes involved in copper homeostasis. Specifically, we focused on Atp7b, a protein essential for copper transport and homeostasis, to determine if Nrf2 directly influenced its transcription.

**FIGURE 7:**
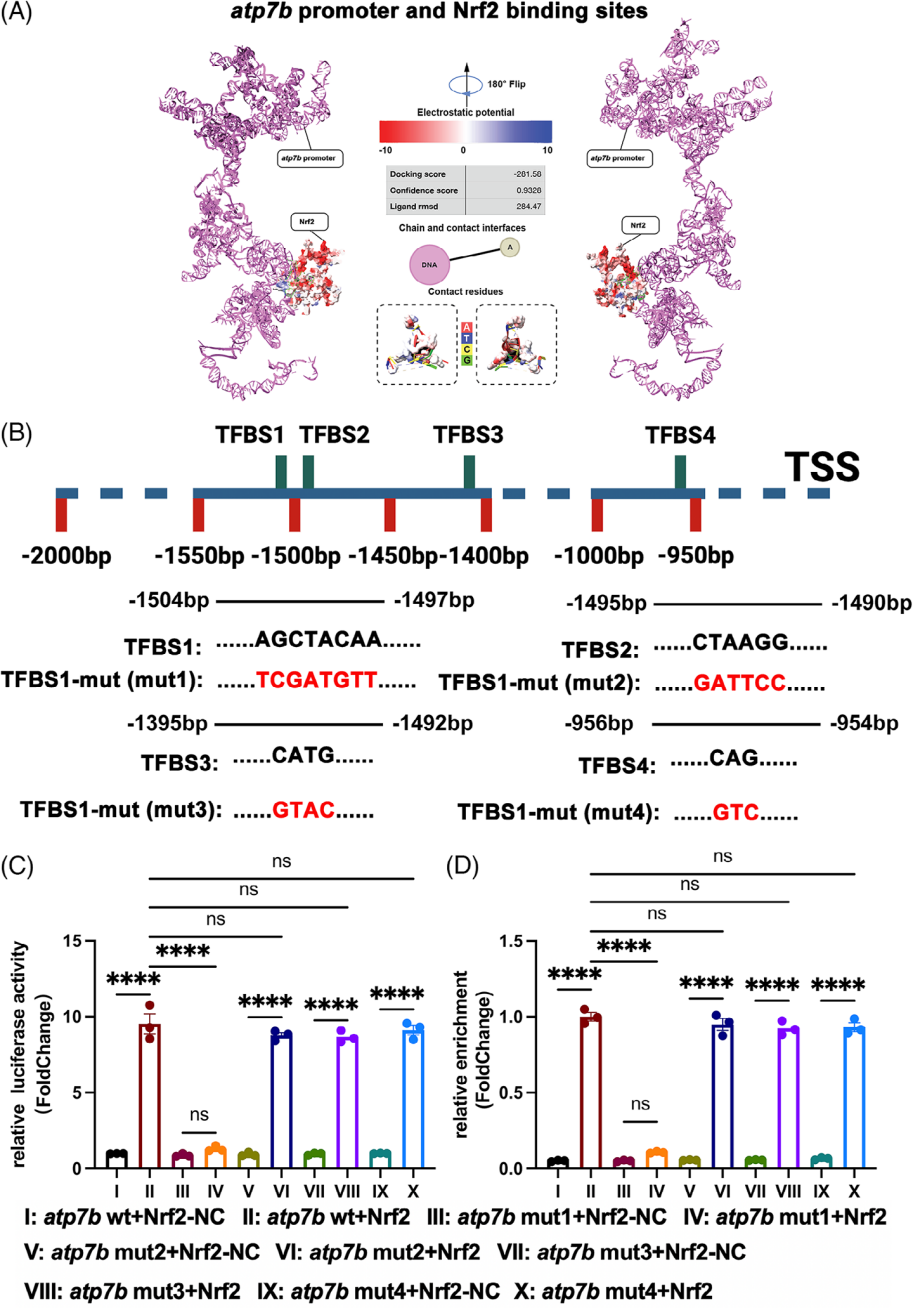
Nrf2 binds to the *atp7b* promoter. (A) Nrf2‐*atp7b* promoter docking: Nrf2 and *atp7b* promoter binding sites. Docking score = −281.58, confidence score = .9328 and ligand rmsd = 284.47. (B) Illustration of the predicted Nrf2 and *atp7b* promoter binding sites. TFBS, transcription factor binding site; TSS, transcription start site. (C) Relative luciferase activity in 293T cells transfected with a plasmid containing wild‐type/mut *atp7b* promoter and Nrf2/Nrf2‐NC (*n* = 3 replicates). (D) Relative enrichment of Nrf2/Nrf2‐NC binding to wild‐type/mut *atp7b* promoter in 293T cells (*n* = 3 replicates). Detailed *p* values for each comparison are provided. Data are presented as the mean ± SEM. One‐way analysis of variance (ANOVA) was used, followed by Tukey's multiple comparisons test. *p < .05, **p < .01, ***p < .001, ****p < .0001, ns, not significant.

Molecular docking was performed to determine the mechanism through which Nrf2 regulates cuproptosis, and that analysis found that Nrf2 could dock with the *atp7b* promoter (Figure 7A). The binding sites of Nrf2 with *atp7b* promoter are shown in Figure 7B. The reliability of these binding sites was investigated by constructing a plasmid with point mutations in the *atp7b* promoter binding sites (*atp7b* mut1, *atp7b* mut2, *atp7*b mut3 and *atp7b* mut4) and transfecting the Nrf2 plasmid into 293T cells. The *atp7b* mut1+Nrf2 group showed significantly decreased luciferase activity compared with the *atp7b*+Nrf2 group. However, the *atp7b* mut2+Nrf2, *atp7b* mut3+Nrf2 and *atp7b* mut4+Nrf2 groups showed no difference compared with the *atp7b*+Nrf2 group (*p* < .0001, Figure 7C). ChIP‐qPCR experiments were performed to validate the binding interactions between Nrf2 and the *atp7b* promoter in a cellular context using 293T cells. The findings indicated that Nrf2 binding to the *atp7b* promoter was significantly diminished in the *atp7b* mut1 group compared to the wild‐type *atp7b* group. However, no notable differences were detected in the *atp7b* mut2, *atp7b* mut3 and *atp7b* mut4 groups. These findings align with our dual‐luciferase reporter assay results and confirm the specificity of the identified binding site in mediating Nrf2‐Atp7b interactions (*p* < .0001, Figure 7D). Thus, TFBS1 is a reliable site for Nrf2 to combine with the *atp7b* promoter.

Our findings revealed a new mechanism whereby Nrf2 regulates cuproptosis and synaptic dysfunction through binding to the *atp7b* promoter, thereby promoting Atp7b transcription. This interaction highlights the critical role of Nrf2 in copper homeostasis and its potential therapeutic application in diseases associated with copper dysregulation.

### 3.6 Overexpression of circSpna2 alleviates cuproptosis and synapse dysfunction via the Keap1/Atp7b signalling axis

Building on our previous findings that Nrf2 binds to the *atp7b* promoter to regulate cuproptosis, we hypothesised that the circSpna2/Keap1/Nrf2/Atp7b signalling axis plays a crucial role in these processes. Given the independent roles of Keap1, Nrf2 and Atp7b in regulating cuproptosis and synaptic dysfunction, we aimed to determine whether the effects of circSpna2 were mediated through this signalling pathway.

The independent roles of Keap1, Nrf2 and Atp7b in regulating cuproptosis and synapse dysfunction were explored in overexpression and knockdown experiments in H_2_O_2_‐treated HT22 cells (Supporting Information Figures 4–8). HT22 cells, derived from mouse hippocampal neurons, are particularly useful for investigating oxidative stress mechanisms and neuroprotective strategies due to their sensitivity to oxidative damage and their ease of manipulation in vitro. Subsequently, we designed rescue experiments to determine if circSpna2 modulated these processes via the Keap1/Nrf2/Atp7b axis. We transfected oe‐circSpna2 with oe‐Keap1 or sh‐circSpna2 with sh‐Keap1, either individually or together.

**FIGURE 8:**
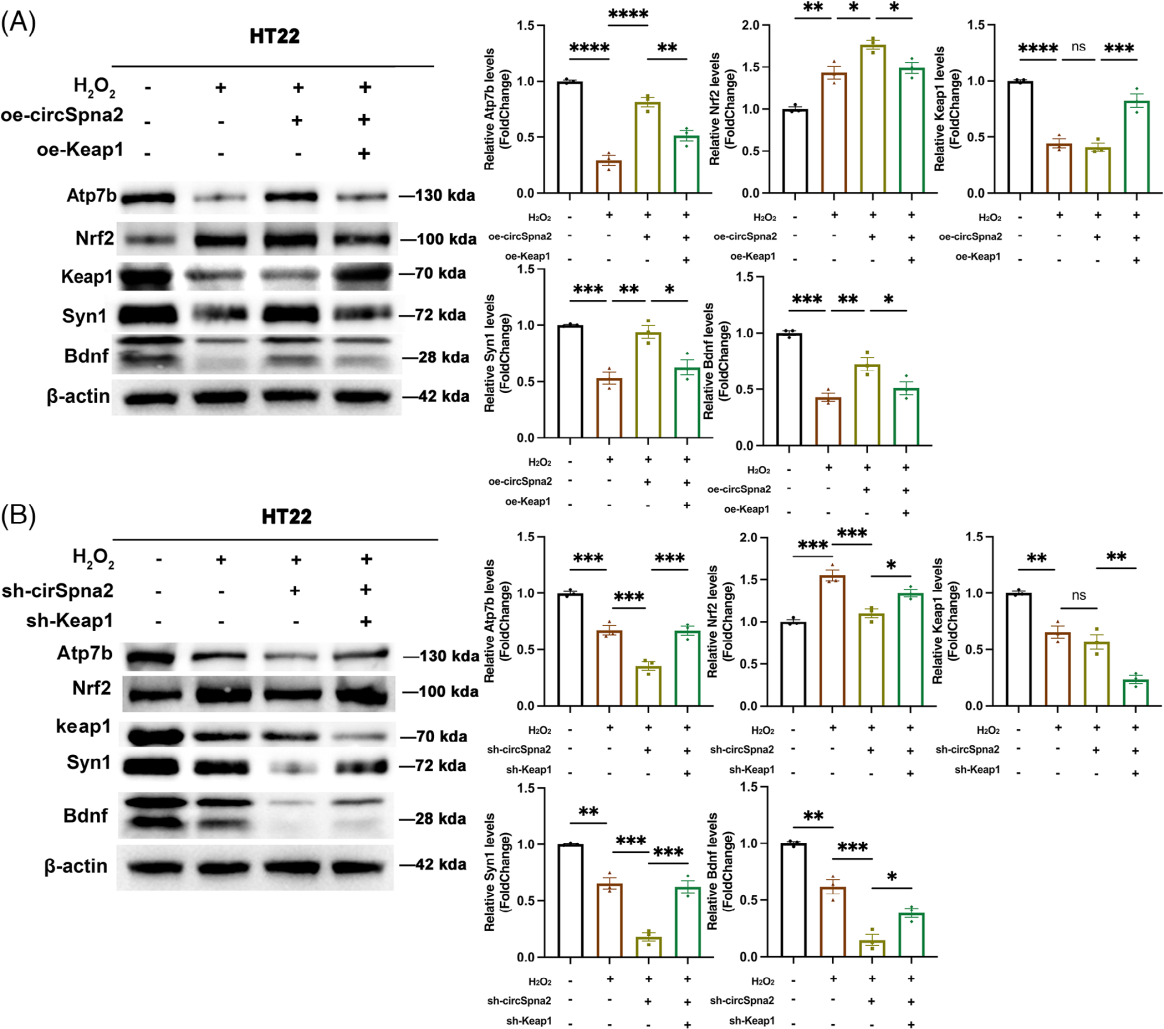
CircSpna2 overexpression regulates the circSpna2/Keap1/Nrf2/Atp7b axis to mitigate H_2_O_2_‐induced synaptic dysfunction and support copper homeostasis in HT22 cells. (A) Western blot analysis of Atp7b, Keap1, Nrf2, Syn1 and Bdnf in HT22 cells transduced with overexpressing circSpna2 (oe‐circSpna2) lentivirus for 7 days, transduced with oe‐NC or oe‐Keap1 plasmid for 48 h, and HT22 cells treated with H_2_O_2_ (600 µmol/L) for 6 h. Transfection with oe‐circSpna2 alone attenuated H_2_O_2_‐induced cuproptosis‐mediated synapse dysfunction, and the expression of Atp7b, Nrf2, Syn1 and Bdnf was upregulated, whereas co‐transfection with oe‐Keap1 reversed these effects. (B) Transfection with sh‐circSpna2 alone aggravated H_2_O_2_‐induced cuproptosis‐mediated synapse dysfunction, and the expression levels of Atp7b, Nrf2, Syn1 and Bdnf were downregulated. Co‐transfection with sh‐Keap1 reversed these effects. Detailed *p* values for each comparison are provided. Data are presented as the mean ± SEM. One‐way analysis of variance (ANOVA) was used, followed by Tukey's multiple comparisons test. *p < .05, **p < .01, ***p < .001, ****p < .0001, ns, not significant.

The findings indicated that oe‐circSpna2 transfection alleviated H_2_O_2_‐induced synapse dysfunction, upregulating Atp7b, Nrf2 and Syn1 expression. Co‐transfection with oe‐Keap1 diminished the benefits of circSpna2 overexpression (Figure 8A). Conversely, transfection with sh‐circSpna2 increased H_2_O_2_‐induced synapse dysfunction, downregulating Atp7b, Nrf2 and Syn1. Co‐transfection with sh‐Keap1 reversed these negative effects (Figure 8B).

**FIGURE 9:**
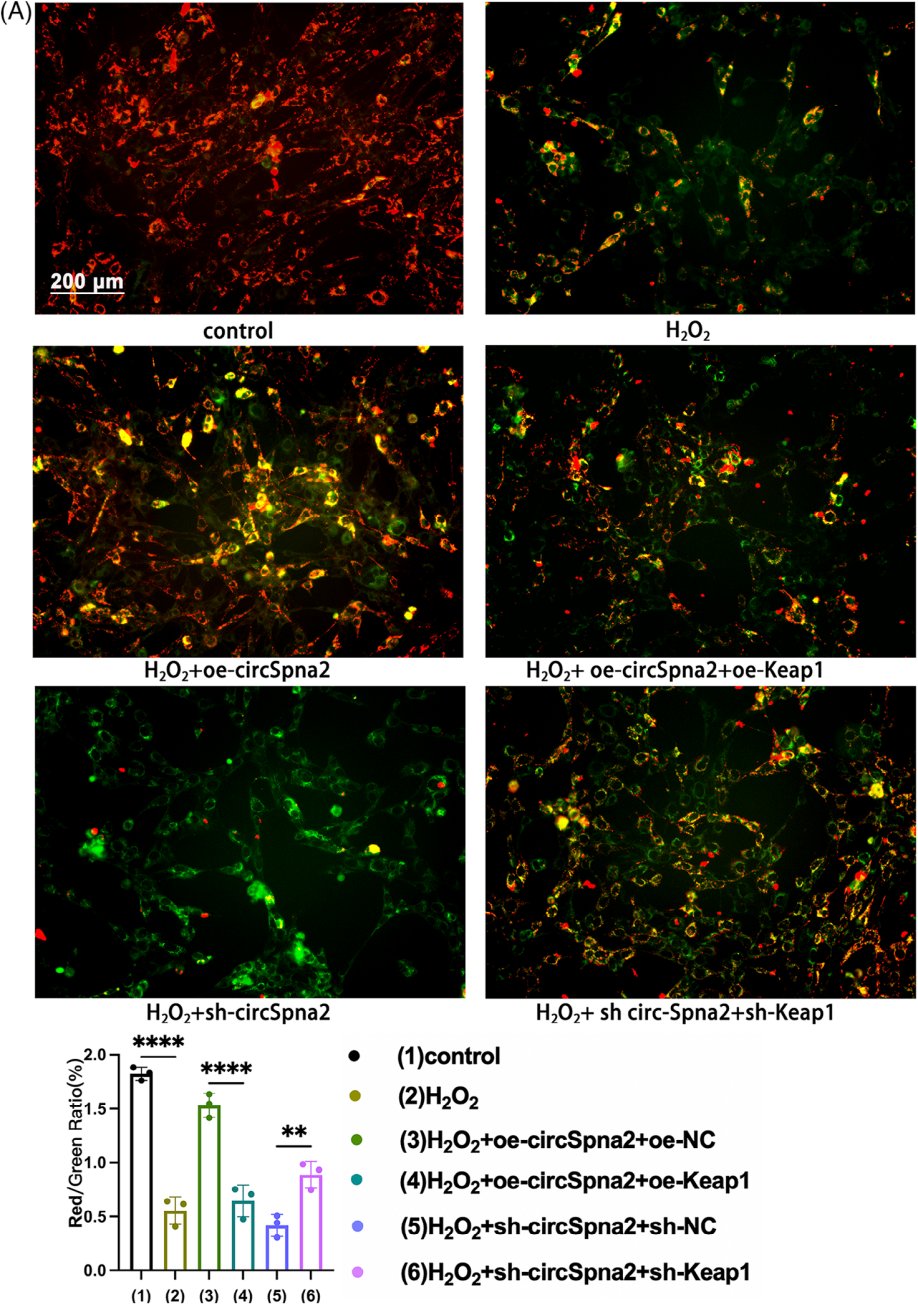
CircSpna2 modulates mitochondrial membrane potential via the circSpna2/Keap1/Nrf2/Atp7b axis in HT22 cells. (A) JC‐1 staining indicated that transduction with the oe‐Keap1 plasmid significantly attenuated increases in mitochondrial membrane potential after overexpressing circSpna2 (oe‐circSpna2) transduction in HT22 cells, where increased mitochondrial membrane potential is indicated by red fluorescence in JC‐1 staining. Conversely, transduction with the sh‐Keap1 plasmid significantly rescued the decreases in mitochondrial membrane potential after sh‐circSpna2 transduction, where decreased mitochondrial membrane potential is indicated by green fluorescence in JC‐1 staining. Detailed *p* values for each comparison are provided. Data are presented as the mean ± SEM. One‐way analysis of variance (ANOVA) was used, followed by Tukey's multiple comparisons test. *p < .05, **p < .01, ***p < .001, ****p < .0001, ns, not significant.

The JC‐1 staining results indicated that transfection with oe‐circSpna2 alone attenuated H_2_O_2_‐induced MMP dysfunction, whereas co‐transfection with oe‐Keap1 reversed these effects in HT22 cells. Transfection with sh‐circSpna2 alone aggravated H_2_O_2_‐induced MMP dysfunction, whereas co‐transfection with sh‐Keap1 reversed these effects (Figure 9A).

**FIGURE 10:**
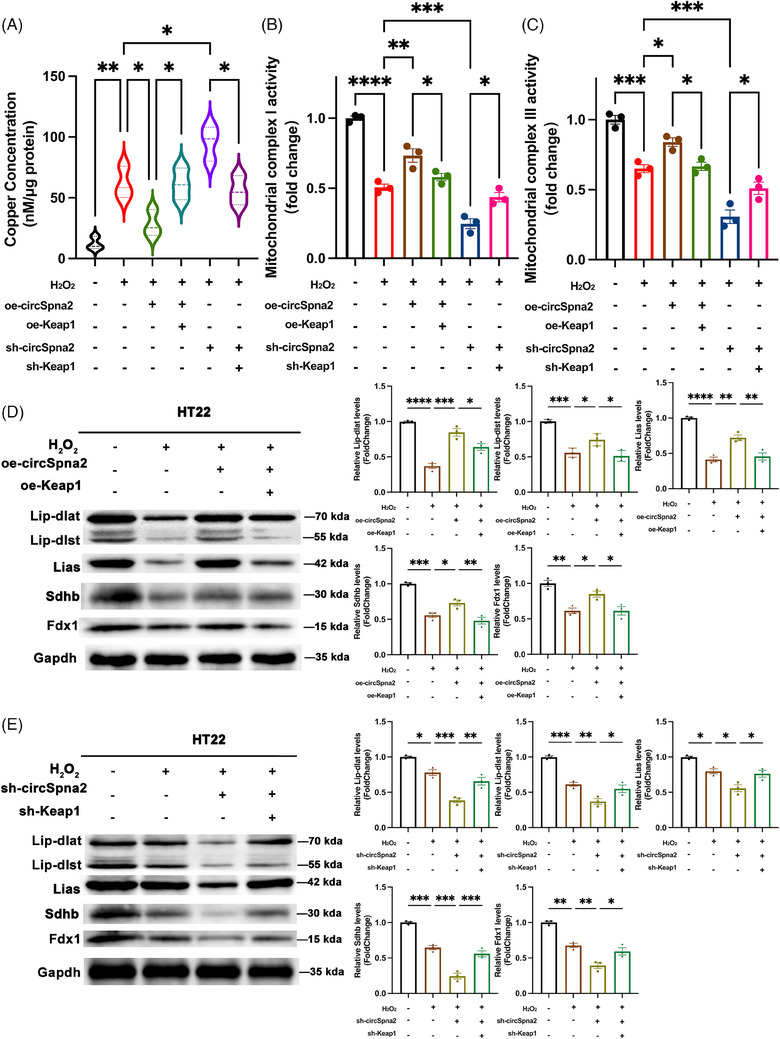
CircSpna2 regulates mitochondrial function and cuproptosis‐related proteins in HT22 cells by modulating the circSpna2/Keap1/Nrf2/Atp7b axis. (A–C) Transduction of HT22 cells with the oe‐Keap1 plasmid significantly reversed overexpressing circSpna2 (oe‐circSpna2)‐induced decreases in copper ion content and increased mitochondrial complex I and III activity. Transduction with sh‐Keap1 plasmid significantly reversed sh‐circSpna2‐induced increases in copper ion content and the decreases in mitochondrial complex I and III activity. (D) Western blot analysis of Lip‐dlat, Lip‐dlst, Lias, Sdhb and Fdx1 in HT22 cells transduced with oe‐circSpna2 lentivirus for 7 days, transduced with oe‐NC or oe‐Keap1 plasmid for 48 h, and H_2_O_2_ (600 µmol/L)‐treated HT22 cells for 6 h showed that transfection with oe‐circSpna2 alone upregulated these protein levels, which were reversed by co‐transfection with oe‐Keap1. (E) Conversely, transfection with sh‐circSpna2 alone downregulated these protein levels, which was reversed by co‐transfection with sh‐Keap1. Detailed *p* values for each comparison are provided. Data are presented as the mean ± SEM. One‐way analysis of variance (ANOVA) was used, followed by Tukey's multiple comparisons test. *p < .05, **p < .01, ***p < .001, ****p < .0001, ns, not significant.

Further analysis showed that H_2_O_2_ treatment significantly increased copper ion levels in HT22 cells and decreased mitochondrial complex I and III activity. The overexpression of circSpna2 significantly reduced intracellular copper ion levels and increased mitochondrial complex I and III activity (Figure 10A–C). However, co‐transfection with oe‐Keap1 plasmid reversed these positive effects. The knockdown of circSpna2 increased copper ion levels and decreased mitochondrial complex I and III activity, but co‐transfection with sh‐Keap1 plasmid rescued these negative effects (Figure 10A–C).

H_2_O_2_ treatment significantly reduced the expression levels of cuproptosis‐related proteins, including Atp7b, Lip‐dlat, Lip‐dlst, Lias, Sdhb and Fdx1. The overexpression of circSpna2 significantly restored the expression levels of these proteins, whereas their reduction was further exacerbated following circSpna2 knockdown. However, co‐transfection with oe‐Keap1 reversed these positive effects, and co‐transfection with sh‐Keap1 rescued the negative effects (Figure 10D,E). These findings highlight the critical role of circSpna2 in regulating copper ion homeostasis and maintaining mitochondrial function.

Our findings indicate that circSpna2 overexpression alleviated cuproptosis and synapse dysfunction via the circSpna2/Keap1/Nrf2/Atp7b signalling axis. This part of the research elucidated the regulatory mechanism of circSpna2 on key proteins involved in copper homeostasis and synaptic integrity, and highlighted the importance of the circSpna2/Keap1/Nrf2/Atp7b axis in mitigating the effects of cuproptosis and maintaining synaptic function, potentially offering new therapeutic strategies for TBI‐induced mood disorders.

## 4 DISCUSSION

**FIGURE 11:**
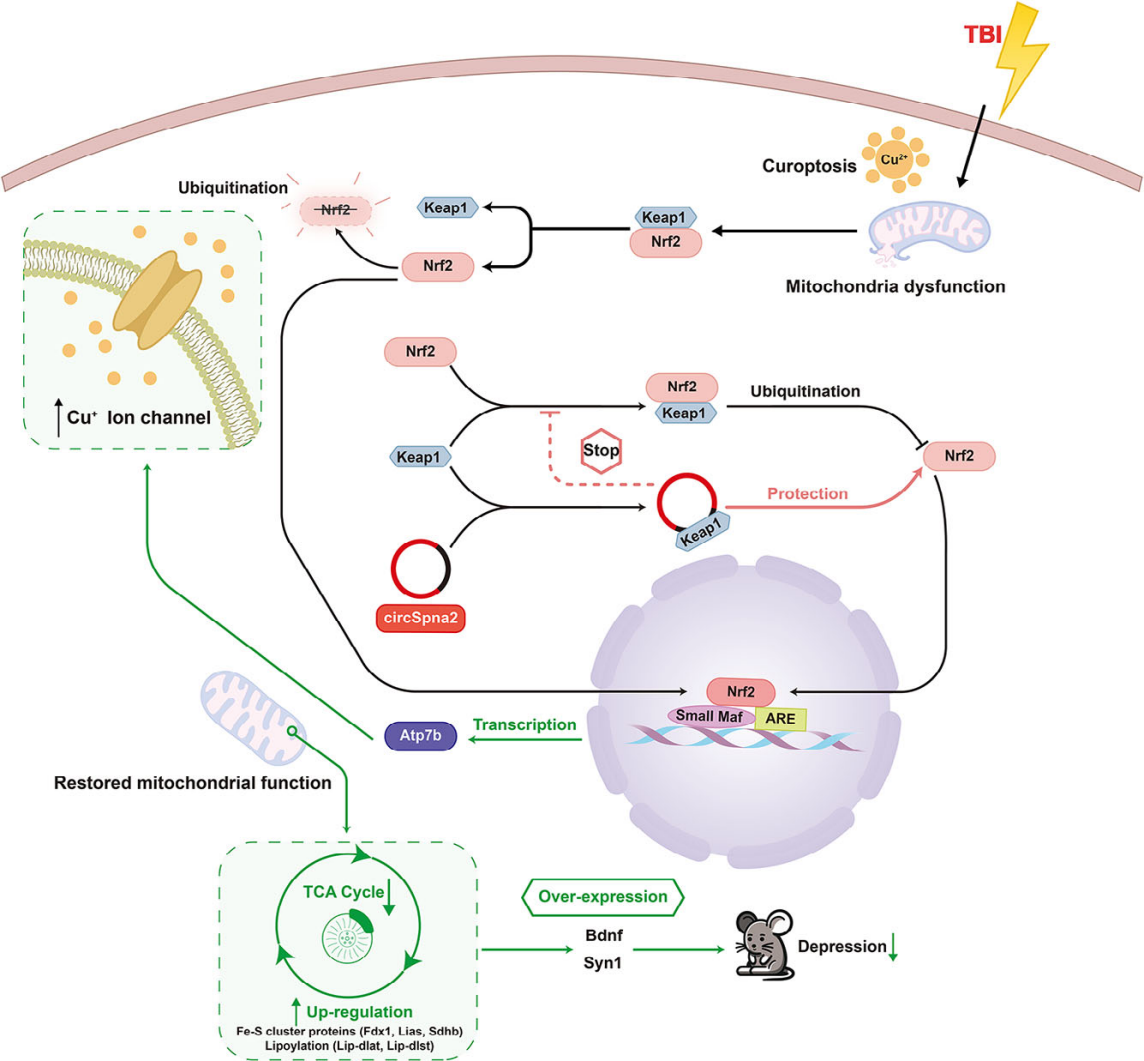
The schematic model shows that circSpna2 alleviates cuproptosis and synapse dysfunction. Traumatic brain injury (TBI) or H_2_O_2_ intervention results in upregulation of Cu^2+^, mitochondrial dysfunction and downregulation of circSpna2. CircSpna2 could adsorb Keap1 as a sponge, which elevates the expression of Nrf2. The upregulate Nrf2 then combines with the *atp7b* promoter to enhance the expression of Atp7b protein, which alleviates cuproptosis. Cuproptosis in neuronal cells also leads to the degradation of Bdnf and Syn1, which impairs neural function. CircSpna2 can be used as a new target for depression after TBI.

To our knowledge, this was the first study to report circSpna2 as a potential biomarker of depression after TBI. In this study, circSpna2 expression decreased in TBI patients and showed a negative correlation with TBI‐related depression. First, mmu_circ_0009804 was selected as the study target based on the bioinformatics analysis. Mmu_circ_0009804 and hsa_circ_0088825 were determined to be 90.6% homologous. The depression scores indicated that hsa_circ_0088825 was associated with depression in TBI patients. Subsequent animal experiments demonstrated that mmu_circ_0009804 was involved in post‐TBI depression and influenced the function of mitochondria, thereby contributing to cuproptosis. This changed the expression of Syn1 and Bdnf, and, ultimately, circSpna2, leading to depression after TBI. H_2_O_2_‐treated HT22 cells were used as a cell model to explore the molecular mechanism due to their origin in mouse hippocampal neurons, which makes them particularly suitable for oxidative stress studies and neuroprotective research. H_2_O_2_ treatment is a widely used method to induce oxidative stress in vitro, mimicking the cellular environment following TBI. This approach allowed us to study the molecular pathways involved in oxidative stress response and cell death mechanisms, such as cuproptosis, in a controlled setting. We found that Keap1 could bind circSpna2 and impact the ubiquitylation of Nrf2, which altered the expression of cuproptosis proteins. These findings suggest that the overexpression of circSpna2 ameliorated TBI‐related depression by elevating the expression levels of Syn1 and Bdnf via the circSpna2/Keap1/Nrf2/Atp7b signalling pathway (Figure 11).

CircRNAs, which are ncRNAs, have attracted recent attention as potential biomarkers of depression.^49^ The study revealed that circPTK2 was decreased in major depressive disorder (MDD) patients, and it might be a miR‐182‐5p sponge influencing depression.^50^ Several studies reported the involvement of circDYM in MDD pathophysiological processes through various signalling axes.^51^, ^52^, ^53^ However, few studies have suggested circRNAs as potential biomarkers of post‐TBI depression. In our study, circSpna2 declined after TBI, and depressive scores (including HAMD, MADRS, SDS and BDI) among TBI patients were associated with plasma hsa_circ_0088825 expression. Recent studies reported that circRNAs were extensively expressed in the human brain, where they constituted a significant portion of the RNA transcriptome. These molecules are involved in various regulatory functions and are dynamically expressed across different brain regions and developmental stages.^54^, ^55^ CircRNAs are known to play roles in synaptic function and neuronal plasticity, making them critical in the context of neurological conditions.^56^ CircSpna2 identified in this study could be a potential biomarker of depression after TBI in the plasma of patients, which physicians could use to recognise patients at a high risk of depression after TBI. Secondly, the techniques of encapsulating RNA fragments by liposomes might deliver exogenous circSpna2, and engineered exosomes might act as the delivery carrier. However, they are still confronted with great challenges, and more clinical trials are needed to examine their efficacy.

Cuprum participates in various biological processes and diseases. The accumulation of copper ions can lead to cell damage and, ultimately, cuproptosis, a novel type of cell death. Cuproptosis has been widely researched in carcinomas^57^, ^58^, ^59^ and studied in other diseases, including ischaemic stroke^60^ and depression.^19^ Increased copper concentrations and toxicity have also been reported in TBI models.^61^ Mitochondrial dysfunction and oxidative stress are essential causes of cuproptosis.^16^ However, few studies have suggested that cuproptosis might play a role in circRNA alterations after TBI. Thus, our study aimed to link cuproptosis with the impact of circSpna2 in post‐TBI depression. Our results first demonstrated that the overexpression of circSpna2 could alleviate depressive‐like behaviours in mice after TBI. We also showed ultrastructural changes in mitochondria and synaptic vesicle numbers. We then showed that the upregulation of circSpna2 could increase the expression levels of Nrf2, Bdnf and Syn1 but not that of Keap1. Nrf2 is reported to be a vital transcription factor in cellular defences against oxidative damage, and the E3 ubiquitin ligase Keap1 might mediate the ubiquitination of Nrf2, as they are both involved in mitochondrial activity and oxidative stress.^62^ The excessive accumulation of copper ions caused by mitochondrial dysregulation could induce cell death, which might trigger depression.^20^, ^63^ Bdnf is a crucial factor affecting hippocampal neurogenesis, and Syn1 is a synapse‐associated protein. Increases in their expression participate in the anti‐depressant process.^64^, ^65^ Subsequently, we showed that upregulating circSpna2 reduced copper ion levels in mice brains and increased the activity of mitochondrial complexes I and III. The expression levels of Atp7b and other cuproptosis‐related markers were also elevated, indicating the alleviation of cuproptosis after oe‐circSpna2. These results are consistent with previous studies of depression and provide a novel viewpoint that circSpna2 overexpression may ameliorate TBI‐related depression by weakening cuproptosis. Notably, redox status is also involved in ferroptosis after TBI, and the antioxidant role of Nrf2 in ferroptosis in TBI has also been extensively studied.^66^, ^67^ The mechanism presented in our study might connect ferroptosis with Nrf2 alterations caused by circSpna2, and represent another mechanism of depression after TBI, which requires further research. Our use of HT22 cells, which are derived from mouse hippocampal neurons and known for their sensitivity to oxidative stress, adds an additional layer of biological relevance. The results observed in HT22 cells showed similar patterns to those in vivo, underscoring the potential therapeutic application of circSpna2 in managing neurodegenerative changes and mood disorders associated with TBI. This detailed correspondence between the in vitro and in vivo models highlights the robustness and translational potential of our findings, reinforcing the hypothesis that circSpna2 plays a crucial role in copper homeostasis and neuroprotection. We noted that Wilson's disease (WD), which is characterised by a copper metabolic disturbance due to inheritable malfunctioning or missing Atp7b protein, can also lead to psychiatric symptoms.^66^ Our study indicated that circSpna2 could regulate the expression of Atp7b to impact cuproptosis and alleviate depression status, also providing a new molecular therapy candidate for WD.

Among their known functions, circRNAs act as miRNA sponges to regulate downstream genes via ce‐RNA mechanisms. Interactions between circRNAs and proteins have received increasing research attention. For example, hsa_circ_0007990 inhibits YBX1 protein to promote breast cancer growth,^67^ while CircPCNXL2 is reported to promote cholangiocarcinoma metastasis by interacting with STRAP.^68^ In the present study, we assumed that Keap1 is a potential target of circSpna2 that regulates the following processes. The bioinformatics analysis, pull‐down and RIP assay results verified the exact binding sites of circSpna2 and Keap1, and Nrf2 expression and ubiquitination levels were influenced by circSpna2 via Keap1. Our results indicated that overexpressing and knocking down Keap1 altered the expression of Nrf2, Syn1, Bdnf, Atp7b and other cuproptosis‐related genes. The results of the rescue experiments provide preliminary evidence that cuproptosis inhibition and the alleviation of TBI‐related depression through circSpna2 were accomplished by binding Keap1 and then regulating Nrf2 expression. Keap1 is a known regulator of Nrf2,^69^ but how Nrf2 affects cuproptosis is unknown. Thus, we researched this in more depth. Atp7b, a copper‐transporting ATPase, plays a vital role in the cellular homeostasis of copper metabolism.^70^ The luciferase activity and ChIP‐qPCR results in the present study showed a reliable binding site of Nrf2 and Atp7b and that overexpressing Atp7b increased the levels of Bdnf and Syn1. This is the first evidence that circSpna2 can ameliorate post‐TBI depression by attenuating cuproptosis through the circSpna2/Keap1/Nrf2/Atp7b signalling axis.

## 5 CONCLUSIONS

In summary, the present study indicated that decreases in circSpna2 after TBI might correlate with TBI‐related depression and suggest a molecular mechanism of circSpna2. Moreover, our findings suggest a novel biomarker for post‐TBI depression that might offer new insight for treatment.

## AUTHOR CONTRIBUTIONS

*Study design and conception*: Mengran Du and Jiayuanyuan Fu. *Animal experiments and TBI model establishment*: Jiayuanyuan Fu, Mengran Du and Jie Zhang. *Plasma collection*: Mengran Du, Jiayuanyuan Fu, Xuekang Huang, Ziyu Zhu, Zhijian Huang, Xin Liu, Qiuhao Tan and Z. B. Liao. *Bioinformatics analysis*: Xuekang Huang and Weilin Tan. *Statistical analysis*: Ziyu Zhu and Lian Liu. *Manuscript draft*: Jiayuanyuan Fu, Mengran Du, Yuan Cheng and Z. B. Liao. All authors approved the final version of the manuscript.

## CONFLICT OF INTEREST STATEMENT

The authors declare no conflicts of interest.

## ETHICS STATEMENT

This study received approval from the Ethics Committee of the First Affiliated Hospital of Chongqing Medical University (No. 2023‐300), with all procedures adhering to the Declaration of Helsinki. All participants or their legally authorized representatives gave written informed consent. All animal experiments were sanctioned by the Chongqing Medical University Animal Care and Use Committee and conducted following the NIH Guide for the Care and Use of Laboratory Animals (NO. IACUC‐CQMU‐2023‐0258).

## Supporting information



Supporting Information

Supporting Information

Supporting Information

Supporting Information

Supporting Information

Supporting Information

Supporting Information

Supporting Information

Supporting Information

Supporting Information

Supporting Information

Supporting Information

Supporting Information

Supporting Information

Supporting Information

## Data Availability

The RNA‐Seq data that support the findings of this study are publicly available in the NCBI Sequence Read Archive (SRA) under the accession number PRJNA725662. This dataset can be accessed at https://www.ncbi.nlm.nih.gov/bioproject/PRJNA725662.
