# Supplementary figures and images for: CircSpna2 attenuates cuproptosis by mediating ubiquitin ligase Keap1 to regulate the Nrf2‐Atp7b signalling axis in depression after traumatic brain injury in a mouse model
Source: Clin Transl Med. 2024 Nov 24;14(11):e70100. doi: 10.1002/ctm2.70100 (PMC11586089; doi:10.1002/ctm2.70100)

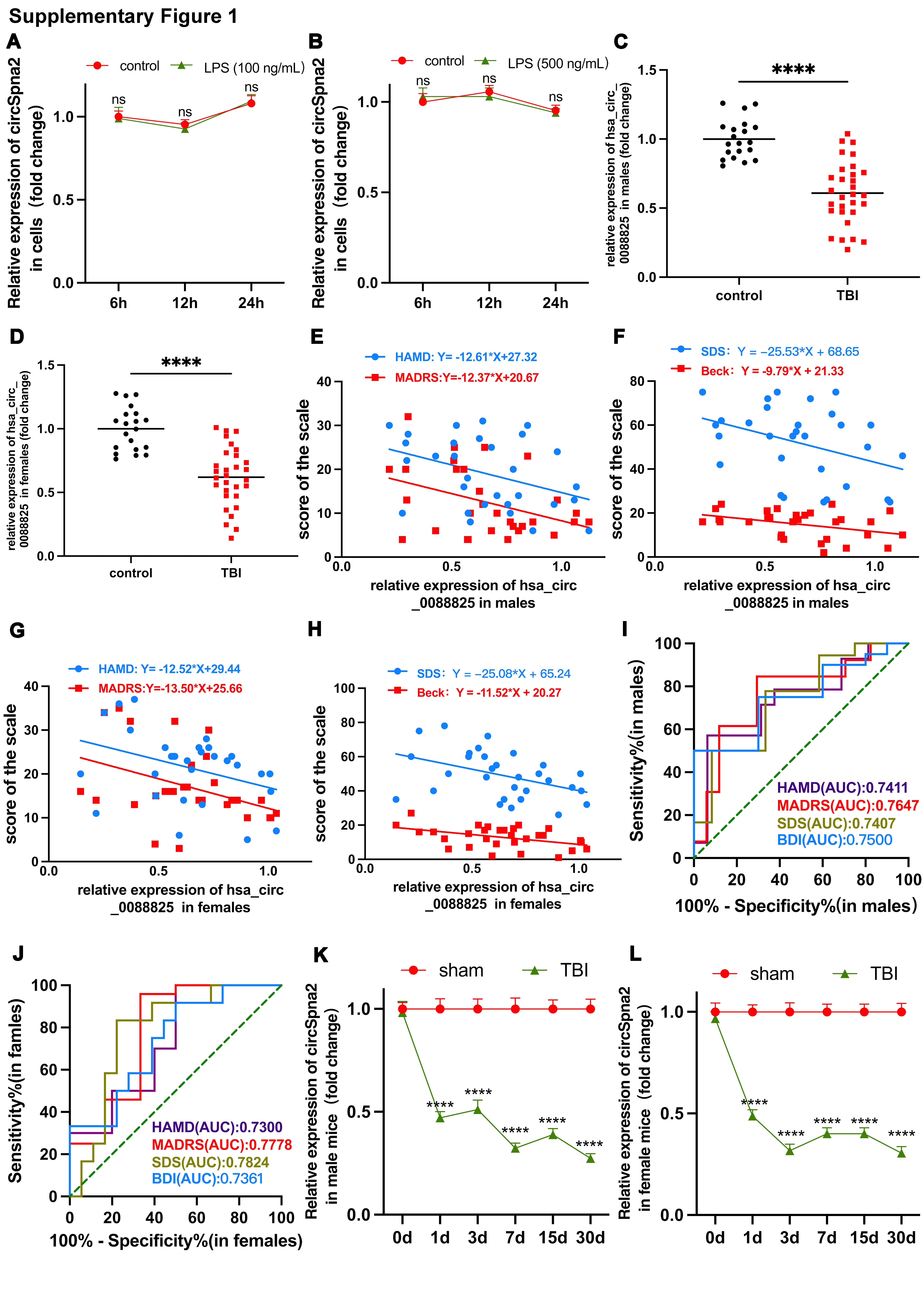

Supplement: Supplementary file 1 — Supporting Information [file CTM2-14-e70100-s015.tif]

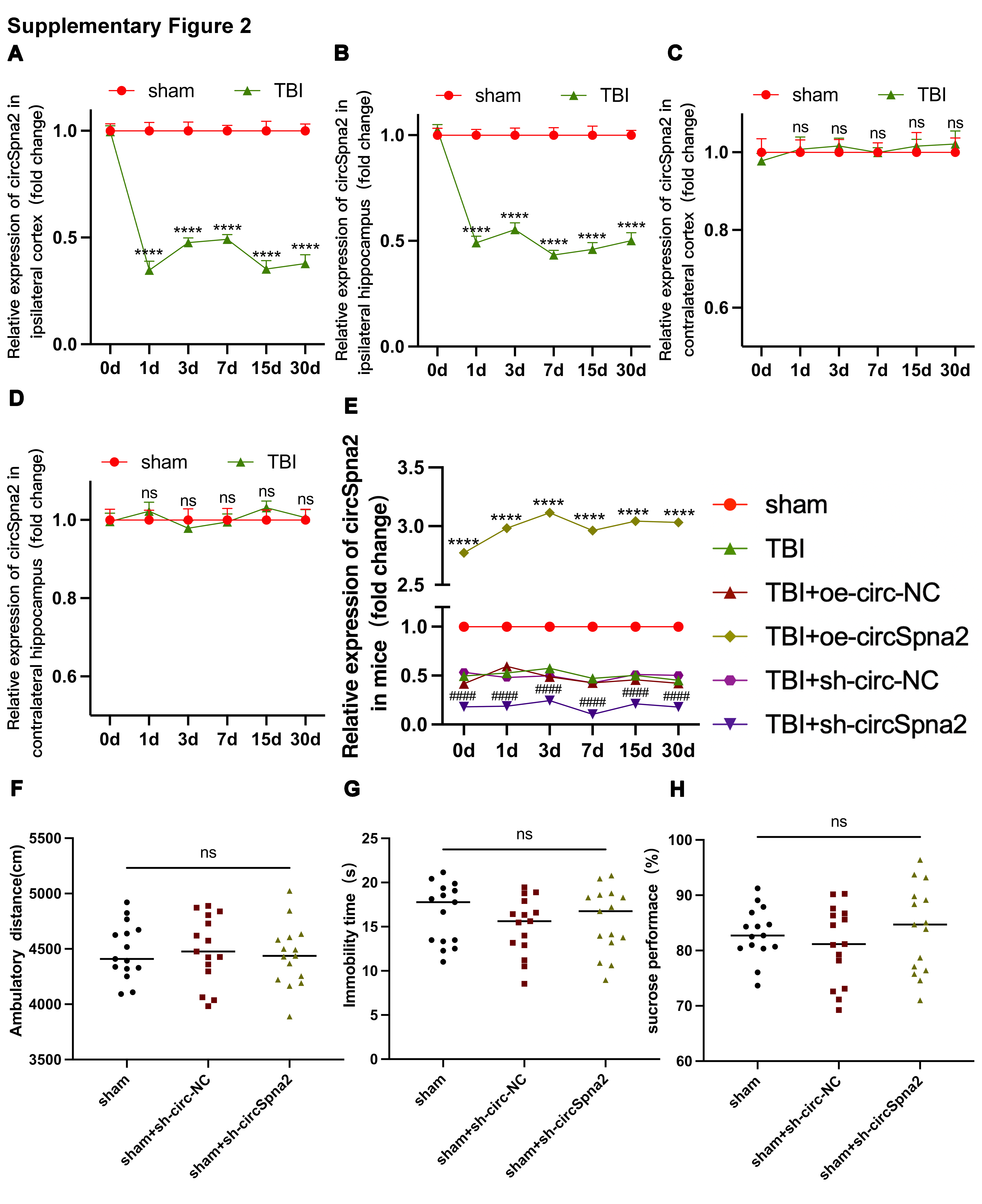

Supplement: Supplementary file 2 — Supporting Information [file CTM2-14-e70100-s013.tif]

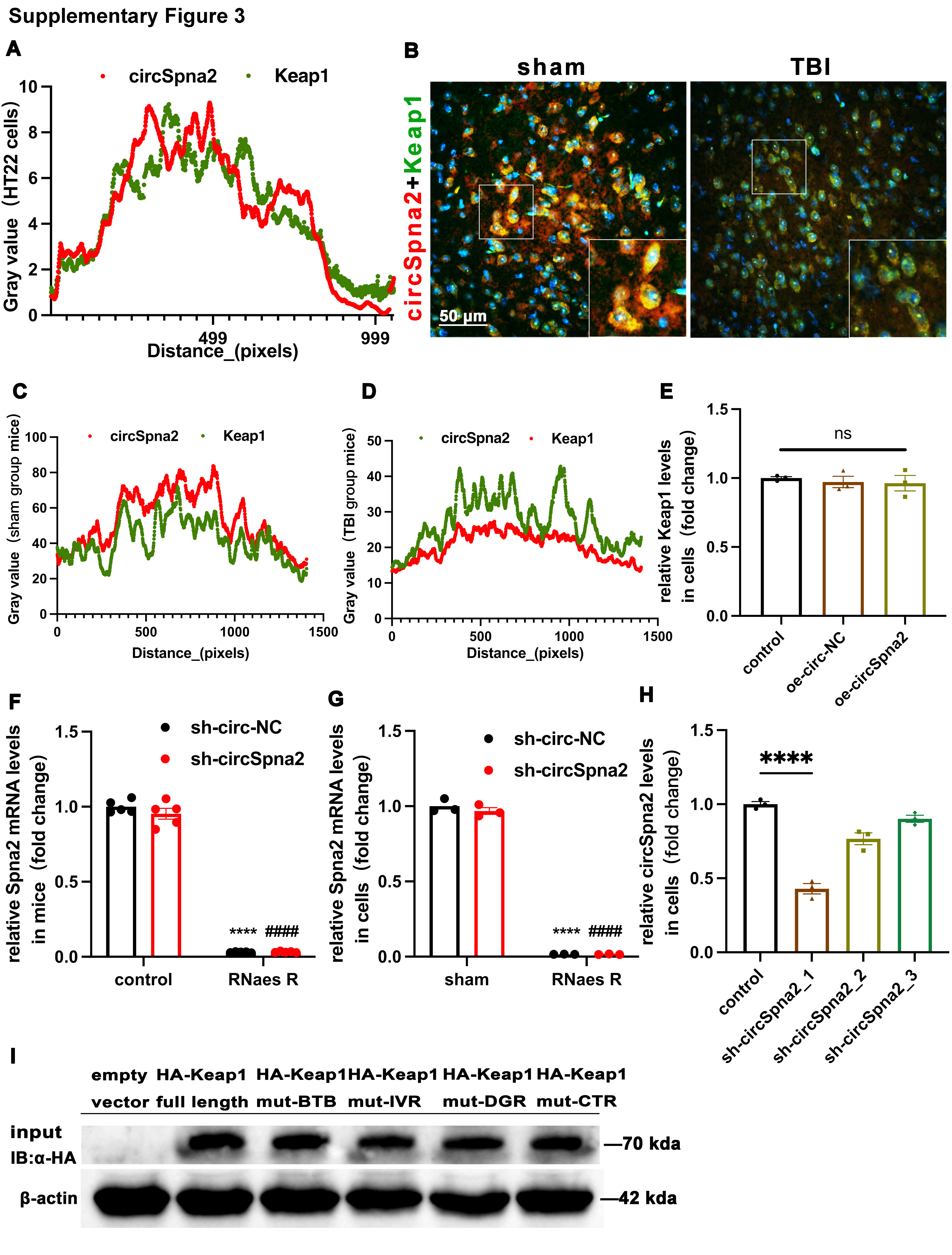

Supplement: Supplementary file 3 — Supporting Information [file CTM2-14-e70100-s009.tif]

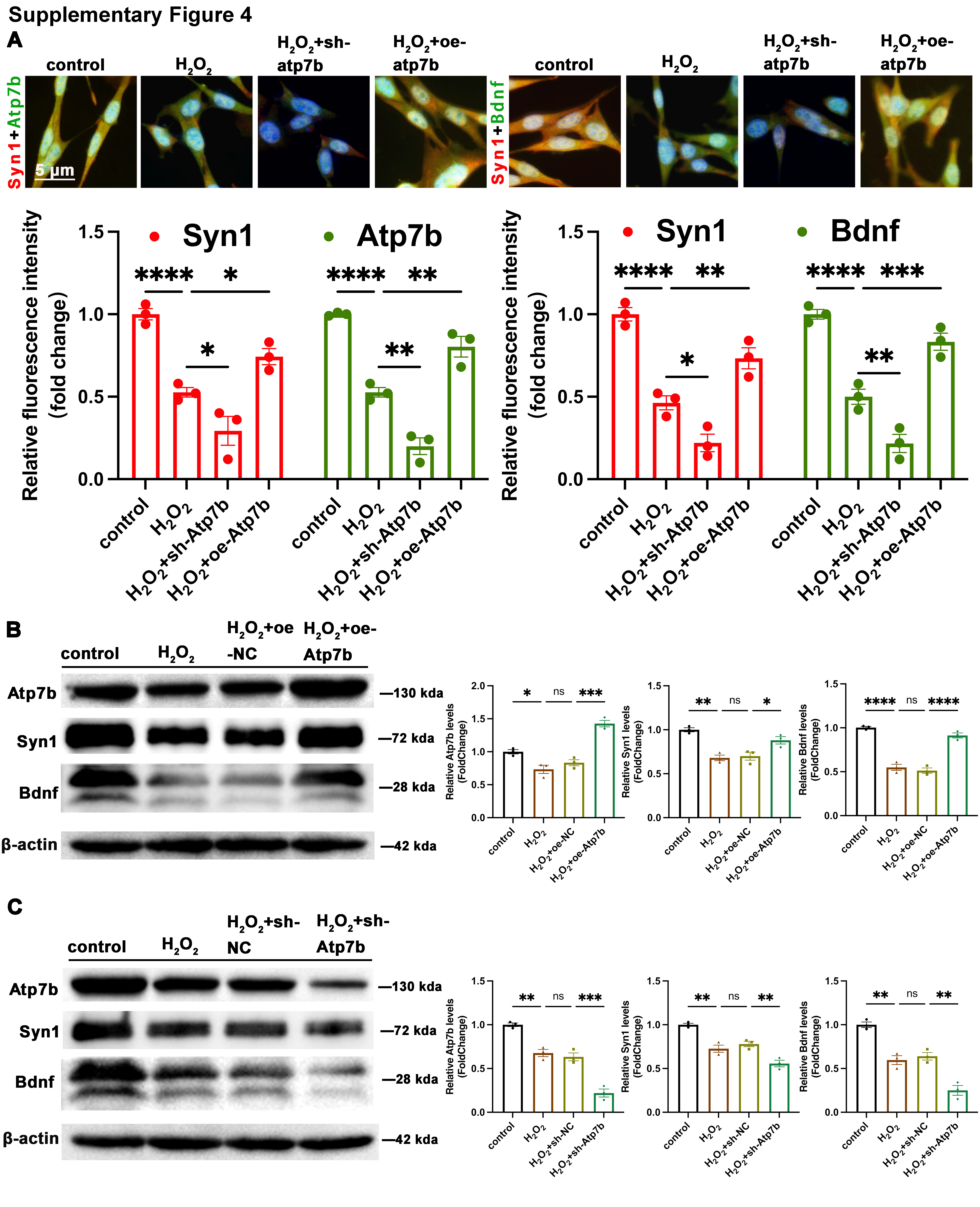

Supplement: Supplementary file 4 — Supporting Information [file CTM2-14-e70100-s007.tif]

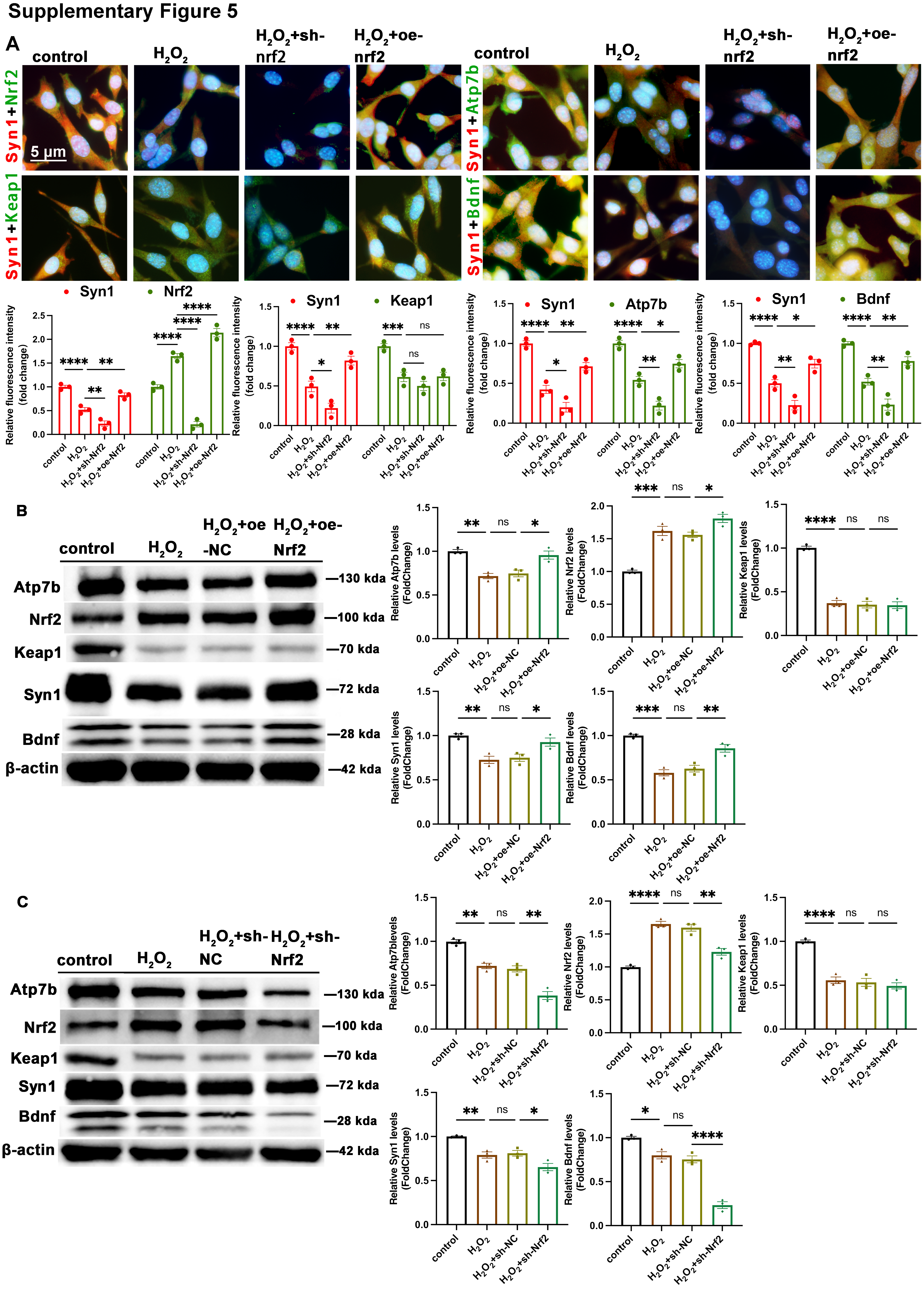

Supplement: Supplementary file 5 — Supporting Information [file CTM2-14-e70100-s012.tif]

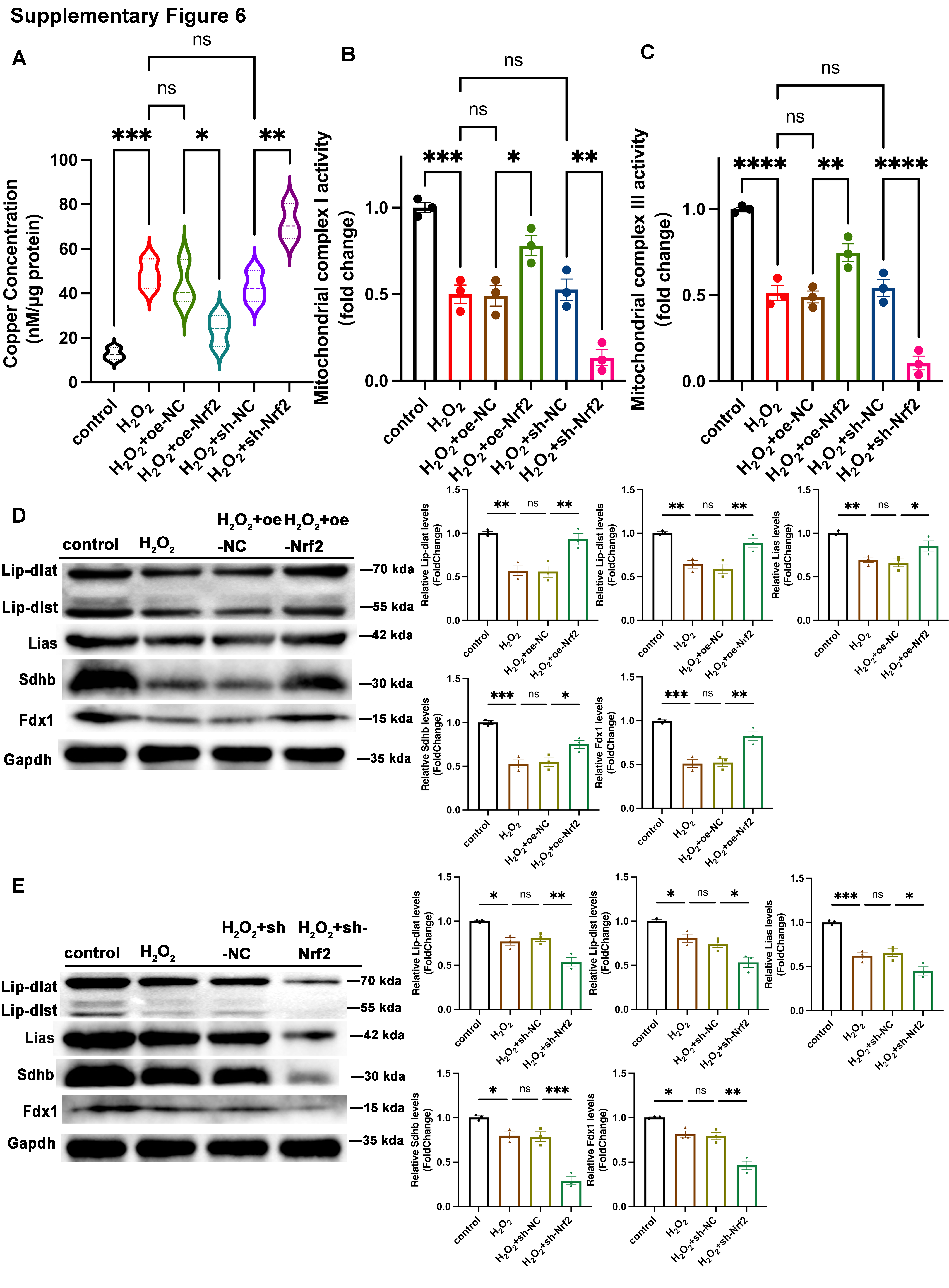

Supplement: Supplementary file 6 — Supporting Information [file CTM2-14-e70100-s002.tif]

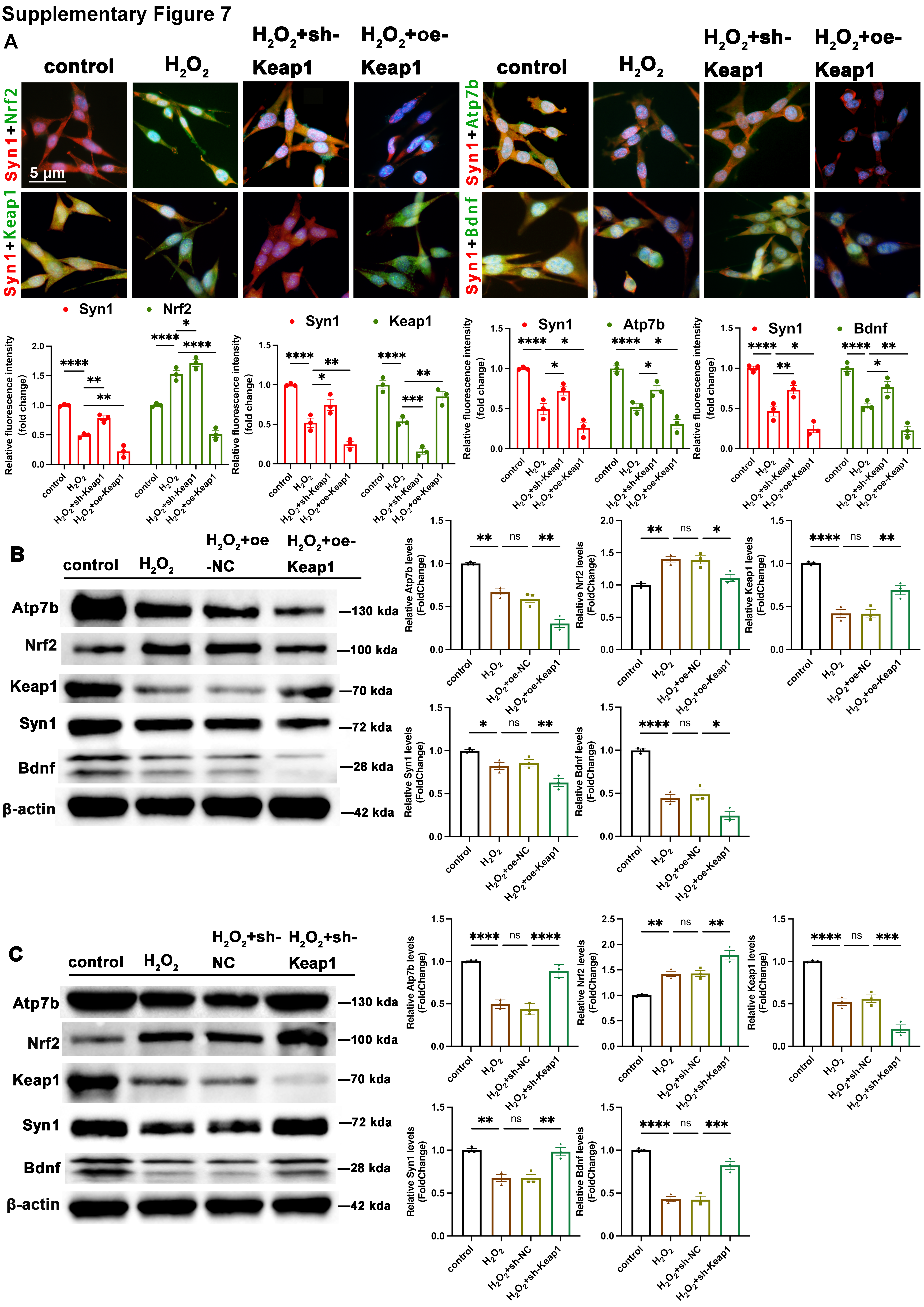

Supplement: Supplementary file 7 — Supporting Information [file CTM2-14-e70100-s004.tif]

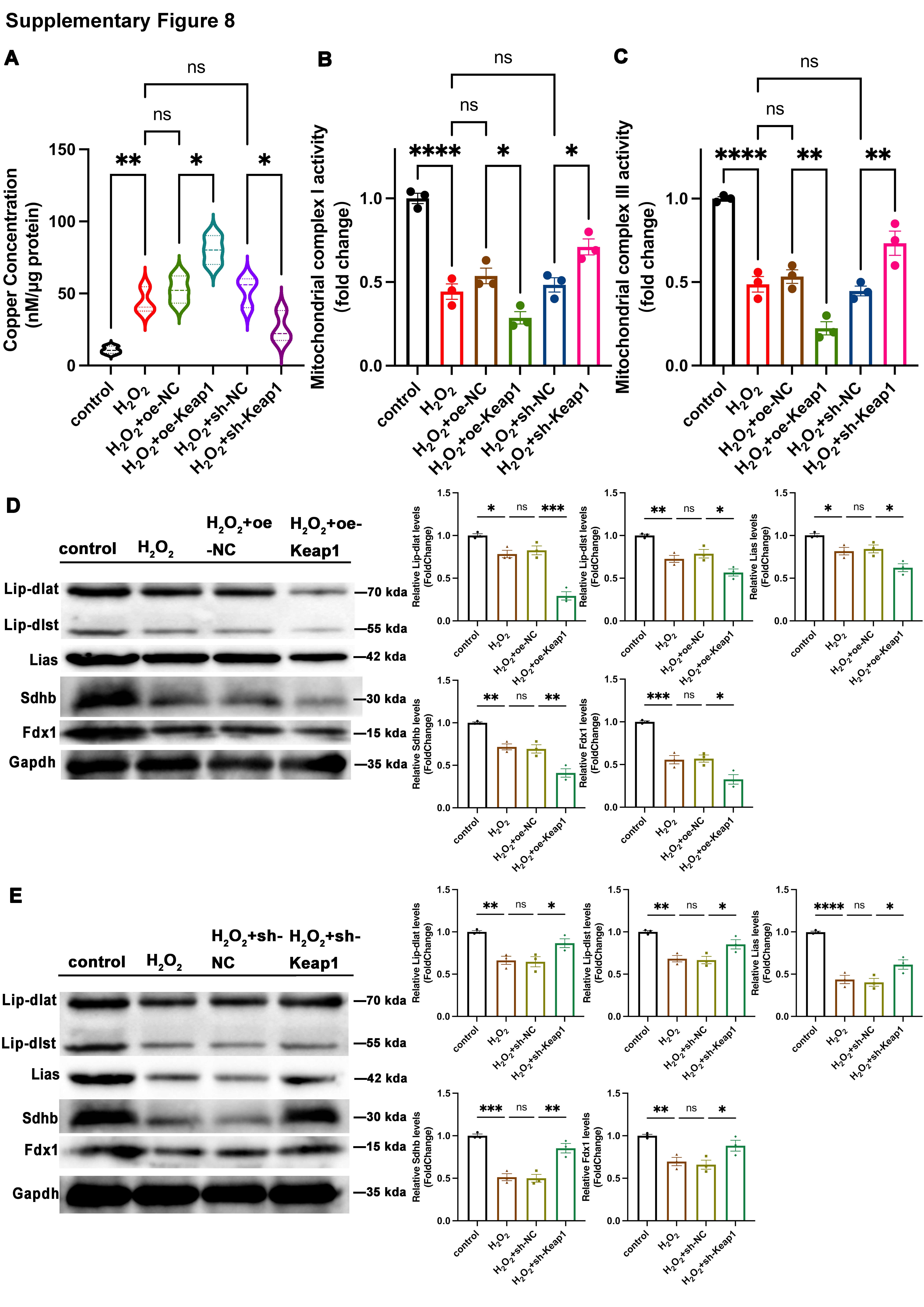

Supplement: Supplementary file 8 — Supporting Information [file CTM2-14-e70100-s008.tif]
