# Supplementary material for: CircSpna2 attenuates cuproptosis by mediating ubiquitin ligase Keap1 to regulate the Nrf2‐Atp7b signalling axis in depression after traumatic brain injury in a mouse model
Source: Clin Transl Med. 2024 Nov 24;14(11):e70100. doi: 10.1002/ctm2.70100 (PMC11586089; doi:10.1002/ctm2.70100)
Supplement: Supplementary file 9 — Supporting Information [file CTM2-14-e70100-s011.docx]

**Supplementary figure legends**

**Supplementary Figure 1. Expression of hsa_circ_0088825 and its correlation with depression scales in TBI patients and mouse models.**

(A) Relative expression of circSpna2 in HT22 cells treated with 100 ng/mL LPS at 6, 12, and 24 hours compared to the control group. No significant changes in circSpna2 expression were observed at any time point (ns: not significant). (B) Relative expression of circSpna2 in HT22 cells treated with 500 ng/mL LPS at 6, 12, and 24 hours compared to the control group. No significant changes in circSpna2 expression were observed across all time points (ns: not significant). (C)Relative expression of hsa_circ_0088825 in the control and TBI groups in males. **** p < 0.0001, two-tailed t-test. (D) Relative expression of hsa_circ_0088825 in the control and TBI groups in females. **** p < 0.0001, two-tailed t-test. (E) Correlation between hsa_circ_0088825 expression and HAMD/MADRS scores in male TBI patients. The regression lines are shown for HAMD (blue) and MADRS (red). Linear equations: HAMD: Y = -12.61*X + 27.32, p = 0.0338 MADRS: Y = -12.37*X + 20.67, p = 0.0306. (F) Correlation between hsa_circ_0088825 expression and SDS/Beck scores in male TBI patients. The regression lines are shown for SDS (blue) and Beck (red). Linear equations: SDS: Y = -25.53*X + 68.65, p = 0.0377, Beck: Y = -9.79*X + 21.33, p = 0.0327. (G) Correlation between hsa_circ_0088825 expression and HAMD/MADRS scores in female TBI patients. The regression lines are shown for HAMD (blue) and MADRS (red). Linear equations: HAMD: Y = -12.52*X + 29.44, p = 0.0433, MADRS: Y = -13.50*X + 25.66, p = 0.0322. (H) Correlation between hsa_circ_0088825 expression and SDS/Beck scores in female TBI patients. The regression lines are shown for SDS (blue) and Beck (red). Linear equations: SDS: Y = -25.08*X + 65.24, p = 0.0165, Beck: Y = -11.52*X + 20.27, p = 0.0137. (I) ROC curves for HAMD, MADRS, SDS, and BDI scales in male TBI patients, showing the AUC values: HAMD: 0.7411, p = 0.0248, MADRS: 0.7647, p = 0.0144, SDS: 0.7407, p = 0.0277, BDI: 0.7500, p = 0.0278. (J) ROC curves for HAMD, MADRS, SDS, and BDI scales in female TBI patients, showing the AUC values: HAMD: 0.7300, p =0.0430, MADRS: 0.7778, p =0.0381, SDS: 0.7824, p =0.0098, BDI: 0.7361, p =0.0308. (K) Relative expression of circSpna2 in male mice at different time points post-TBI. **** p < 0.0001 compared to the sham group, two-tailed t-test. (L) Relative expression of circSpna2 in female mice at different time points post-TBI. **** p < 0.0001 compared to the sham group, two-tailed t-test. Detailed p-values for each comparison are provided. All data are presented as the mean ± SEM. * p < 0.05, ** p < 0.01, *** p < 0.001, **** p < 0.0001, ns: not significant.

**Supplementary Figure 2. The effect of circSpna2 knockdown on depressive-like behaviors in wild-type mice.**

(A) Relative expression of circSpna2 in the ipsilateral cortex of TBI mice compared to the sham group. qRT-PCR analysis revealed a significant downregulation of circSpna2 in the ipsilateral cortex at 1, 3, 7, 15, and 30 days post-TBI. (B) Relative expression of circSpna2 in the ipsilateral hippocampus of TBI mice compared to the sham group. A persistent downregulation of circSpna2 was observed at all time points post-TBI. (C) Relative expression of circSpna2 in the contralateral cortex. No significant changes in circSpna2 expression were observed in the contralateral cortex at any time point compared to sham controls. (D) Relative expression of circSpna2 in the contralateral hippocampus. Similar to the contralateral cortex, no significant changes were detected in the contralateral hippocampus across the time points studied. (E) Relative expression of circSpna2 was measured by qRT-PCR in different treatment groups (sham, TBI, TBI+oe-circ-NC, TBI+oe-circSpna2, TBI+sh-circ-NC, and TBI+sh-circSpna2) at 0d, 1d, 3d, 7d, 15d, and 30d post-TBI. In the TBI+oe-circSpna2 group, circSpna2 expression was significantly upregulated at all time points compared to the control groups(TBI+oe-circ-NC) , **** p < 0.0001. Conversely, circSpna2 expression was markedly downregulated in the TBI+sh-circSpna2 group compared to the control groups(TBI+sh-circ-NC), #### p < 0.0001. No significant changes in expression were observed in the sham, TBI, TBI+oe-circ-NC, and TBI+sh-circ-NC groups. (F) Ambulatory distance measured in the open field test (OFT). No significant difference was observed between the sham group, sham+sh-circ-NC group, and sham+sh-circSpna2 group, indicating that circSpna2 knockdown did not affect locomotor activity in wild-type mice (n = 15 per group). (G) Immobility time measured in the tail suspension test (TST). No significant difference was observed in immobility time between the sham group, sham+sh-circ-NC group, and sham+sh-circSpna2 group, indicating that circSpna2 knockdown did not induce depressive-like behaviors in wild-type mice (n = 15 per group). (H) Sucrose preference percentage measured in the sucrose preference test (SPT). There was no significant difference in sucrose preference between the sham group, sham+sh-circ-NC group, and sham+sh-circSpna2 group, indicating that circSpna2 knockdown did not affect hedonic behaviors in wild-type mice (n = 15 per group). Data are presented as mean ± SEM, and statistical analysis was performed using one-way ANOVA (ns, not significant). * p < 0.05, ** p < 0.01, *** p < 0.001, **** p < 0.0001, ns: not significant.

**Supplementary Figure 3. Colocalization and effects of circSpna2 on Keap1.**

(A) Colocalization of circSpna2 and Keap1 in HT22 cells, with the corresponding gray value distribution along the distance. (B) Representative immunofluorescence images showing colocalization of circSpna2 (red) and Keap1 (green) in brain slices from both sham and TBI mice. (C) Gray value distribution of circSpna2 and Keap1 in brain tissue from sham mice. (D) Gray value distribution of circSpna2 and Keap1 in brain tissue from TBI mice. (E) Relative Keap1 levels in cells treated with control, oe-circ-NC, or oe-circSpna2. Data are presented as fold change relative to control. ns indicates no significant difference, two-tailed t-test. (F) Relative Spna2 mRNA levels in mice treated with sh-circ-NC or sh-circSpna2 under sham and RNAes R conditions. Data are presented as fold change relative to sham. **** p < 0.0001 versus sham; #### p < 0.0001 versus RNAes R, two-tailed t-test. (G) Relative Spna2 mRNA levels in cells treated with sh-circ-NC or sh-circSpna2 under control and RNAes R conditions. Data are presented as fold change relative to control. **** p < 0.0001 versus control; #### p < 0.0001 versus RNAes R, two-tailed t-test. (H) Relative circSpna2 levels in cells treated with control, sh-circSpna2_1, sh-circSpna2_2, or sh-circSpna2_3. (I) Western blot analysis of HA-tagged Keap1 and its mutants (mut-BTB, mut-IVR, mut-DGR, and mut-CTR) in HT22 cells. Cell lysates were analyzed using anti-HA antibody to confirm the expression of full-length Keap1 and its mutants. β-actin was used as a loading control. Detailed p-values for each comparison are provided. Data are presented as mean ± SEM. *p < 0.05, **p < 0.01, ***p < 0.001, ****p < 0.0001, ns: not significant.

**Supplementary Fig. 4 Atp7b alleviates synapse dysfunction.**

(A) Co-localization of Syn1 with Bdnf or Atp7b was detected by double immunofluorescence in the control, H_2_O_2_, H_2_O_2_+oe-Atp7b, and H_2_O_2_+sh-Atp7b groups. (B) Western blot analysis of Atp7b, Bdnf, and Syn1 expression in HT22 cells treated with H_2_O_2_  (600 µmol/L) for 6 h after Atp7b overexpression. Transfection with oe-Atp7b upregulated the expression of Atp7b, Bdnf, and Syn1 compared to H_2_O_2_ treatment. (C) Western blot analysis of Atp7b, Bdnf, and Syn1 expression in HT22 cells treated with H_2_O_2_ (600 µmol/L) for 6 h after Atp7b knockdown. Transfection with sh-Atp7b downregulated the expression of Atp7b, Bdnf, and Syn1 compared to H_2_O_2_ treatment. Statistical analysis revealed significant differences in the expression levels of Atp7b, Bdnf, and Syn1 between the control and H_2_O_2_ groups, as well as between H_2_O_2_+oe-Atp7b and H_2_O_2_+oe-NC, and H_2_O_2_+sh-Atp7b and H_2_O_2_+sh-NC groups. Detailed p-values for each comparison are provided. Data are presented as mean ± SEM. One-way ANOVA followed by Tukey's multiple comparisons test was used. *p < 0.05, **p < 0.01, ***p < 0.001, ****p < 0.0001, ns: not significant.

**Supplementary Fig. 5 Nrf2 regulates Atp7b to alleviate synapse dysfunction.**

(A) Co-localization of Syn1 with Nrf2, Keap1, Bdnf, or Atp7b was detected by double immunofluorescence in the control, H_2_O_2_, H_2_O_2_+oe-Nrf2, and H_2_O_2_+sh-Nrf2 groups. (B) Western blot analysis of Atp7b, Nrf2, Keap1, Bdnf, and Syn1 expression in HT22 cells treated with H_2_O_2_ (600 µmol/L) for 6 h after Nrf2 overexpression. Transfection with oe-Nrf2 upregulated the expression of Atp7b, Nrf2, Bdnf, and Syn1 compared to H_2_O_2_ treatment. (C) Western blot analysis of Atp7b, Nrf2, Keap1, Bdnf, and Syn1 expression in HT22 cells treated with H_2_O_2_ (600 µmol/L) for 6 h after Nrf2 knockdown. Transfection with sh-Nrf2 downregulated the expression of Atp7b, Nrf2, Bdnf, and Syn1 compared to H_2_O_2_ treatment. Detailed p-values for each comparison are provided. Data are presented as the mean ± SEM. One-way ANOVA was used, followed by Tukey's multiple comparisons test. *p < 0.05, **p < 0.01, ***p < 0.001, ****p < 0.0001, ns: not significant.

**Supplementary Fig. 6 Nrf2 regulates Atp7b to alleviate cuproptosis.**

(A) Copper ion concentration in HT22 cells treated with H_2_O_2_ (600 µmol/L) for 6 h after Nrf2 overexpression or knockdown. Nrf2 overexpression decreased copper ion levels, while Nrf2 knockdown increased copper ion levels compared to H_2_O_2_ treatment alone. (B) Mitochondrial complex I activity in HT22 cells treated with H_2_O_2_ (600 µmol/L) for 6 h after Nrf2 overexpression or knockdown. Nrf2 overexpression increased mitochondrial complex I activity, while Nrf2 knockdown decreased mitochondrial complex I activity compared to H_2_O_2_ treatment. (C) Mitochondrial complex III activity in HT22 cells treated with H_2_O_2_ (600 µmol/L) for 6 h after Nrf2 overexpression or knockdown. Nrf2 overexpression increased mitochondrial complex III activity, while Nrf2 knockdown decreased mitochondrial complex III activity compared to H_2_O_2_ treatment. (D) Western blot analysis of Lip-dlat, Lip-dlst, Lias, Sdhb, and Fdx1 expression in HT22 cells treated with H_2_O_2_ (600 µmol/L) for 6 h after Nrf2 overexpression. Transfection with oe-Nrf2 upregulated these protein levels compared to H_2_O_2_ treatment. (E) Western blot analysis of Lip-dlat, Lip-dlst, Lias, Sdhb, and Fdx1 expression in HT22 cells treated with H_2_O_2_ (600 µmol/L) for 6 h after Nrf2 knockdown. Transfection with sh-Nrf2 downregulated these protein levels compared to H_2_O_2_ treatment. Statistical analysis revealed significant differences in the expression levels of Lip-dlat, Lip-dlst, Lias, Sdhb, and Fdx1 between the different treatment groups. Detailed p-values for each comparison are provided. Data are presented as mean ± SEM. One-way ANOVA followed by Tukey's multiple comparisons test was used. *p < 0.05, **p < 0.01, ***p < 0.001, ****p < 0.0001, ns: not significant.

**Supplementary Fig. 7 Keap1 regulates the Nrf2/Atp7b axis to alleviate synapse dysfunction.**

(A) Co-localization of Syn1 with Nrf2, Keap1, Bdnf, or Atp7b was detected by double immunofluorescence in the control, H_2_O_2_, H_2_O_2_+oe-Keap1, and H_2_O_2_+sh-Keap1 groups. (B) Western blot analysis of Atp7b, Nrf2, Keap1, Bdnf, and Syn1 expression in HT22 cells treated with H_2_O_2_ (600 µmol/L) for 6 h after Keap1 overexpression. Overexpression of Keap1 downregulated the expression of Atp7b, Nrf2, Bdnf, and Syn1 compared to H_2_O_2_ treatment. (C) Western blot analysis of Atp7b, Nrf2, Keap1, Bdnf, and Syn1 expression in HT22 cells treated with H_2_O_2_ (600 µmol/L) for 6 h after Keap1 knockdown. Knockdown of Keap1 upregulated the expression of Atp7b, Nrf2, Bdnf, and Syn1 compared to H_2_O_2_ treatment. Detailed p-values for each comparison are provided. Data are presented as mean ± SEM. One-way ANOVA followed by Tukey's multiple comparisons test was used. *p < 0.05, **p < 0.01, ***p < 0.001, ****p < 0.0001, ns: not significant.

**Supplementary Fig. 8 Keap1 regulates the Nrf2/Atp7b to alleviate cuproptosis.**

(A) Copper ion concentration in HT22 cells treated with H_2_O_2_ (600 µmol/L) for 6 h after Keap1 overexpression or knockdown. Overexpression of Keap1 increased copper ion levels, while knockdown of Keap1 decreased copper ion levels compared to H_2_O_2_ treatment. (B) Mitochondrial complex I activity in HT22 cells treated with H_2_O_2_ (600 µmol/L) for 6 h after Keap1 overexpression or knockdown. Overexpression of Keap1 decreased mitochondrial complex I activity, while knockdown of Keap1 increased mitochondrial complex I activity compared to H_2_O_2_ treatment. (C) Mitochondrial complex III activity in HT22 cells treated with H_2_O_2_ (600 µmol/L) for 6 h after Keap1 overexpression or knockdown. Overexpression of Keap1 decreased mitochondrial complex III activity, while knockdown of Keap1 increased mitochondrial complex III activity compared to H_2_O_2_ treatment. (D) Western blot analysis of Lip-dlat, Lip-dlst, Lias, Sdhb, and Fdx1 expression in HT22 cells treated with H_2_O_2_ (600 µmol/L) for 6 h after Keap1 overexpression. Overexpression of Keap1 downregulated these protein levels compared to H_2_O_2_ treatment. (E) Western blot analysis of Lip-dlat, Lip-dlst, Lias, Sdhb, and Fdx1 expression in HT22 cells treated with H_2_O_2_ (600 µmol/L) for 6 h after Keap1 knockdown. Knockdown of Keap1 upregulated these protein levels compared to H_2_O_2_ treatment. Statistical analysis revealed significant differences in the expression levels of Lip-dlat, Lip-dlst, Lias, Sdhb, and Fdx1 between the different treatment groups. Detailed p-values for each comparison are provided. Data are presented as mean ± SEM. One-way ANOVA followed by Tukey's multiple comparisons test was used. *p < 0.05, **p < 0.01, ***p < 0.001, ****p < 0.0001, ns: not significant.
