# Supplementary material for: CircSpna2 attenuates cuproptosis by mediating ubiquitin ligase Keap1 to regulate the Nrf2‐Atp7b signalling axis in depression after traumatic brain injury in a mouse model
Source: Clin Transl Med. 2024 Nov 24;14(11):e70100. doi: 10.1002/ctm2.70100 (PMC11586089; doi:10.1002/ctm2.70100)
Supplement: Supplementary file 10 — Supporting Information [file CTM2-14-e70100-s005.docx]

**Supplementary Table 1.** Clinical data from control and TBI cases.

| **NO.** | **Age（years）** | **gender** | **time after TBI** | **Contusion site** | **GCS** | **GOS** |
| --- | --- | --- | --- | --- | --- | --- |
| **1** | **32** | **M** | **NA** | **NA** | **15** | **5** |
| **2** | **44** | **F** | **NA** | **NA** | **15** | **5** |
| **3** | **46** | **F** | **NA** | **NA** | **15** | **5** |
| **4** | **42** | **M** | **8h** | **Frontotemporal(LT)** | **8** | **3** |
| **5** | **39** | **F** | **14h** | **Frontal(LT)** | **7** | **3** |
| **6** | **47** | **F** | **12h** | **Frontal(Bilateral)** | **7** | **4** |
| **7** | **36** | **M** | **17h** | **Temporal(RT)** | **8** | **4** |
| **8** | **51** | **M** | **11h** | **Frontal(Bilateral)** | **9** | **3** |
| **9** | **38** | **F** | **14h** | **Frontotemporal(Bilateral)** | **7** | **4** |

Notes:

GCS: Glasgow Coma Scale

GOS: Glasgow Outcome Scale
