# Supplementary material for: CircSpna2 attenuates cuproptosis by mediating ubiquitin ligase Keap1 to regulate the Nrf2‐Atp7b signalling axis in depression after traumatic brain injury in a mouse model
Source: Clin Transl Med. 2024 Nov 24;14(11):e70100. doi: 10.1002/ctm2.70100 (PMC11586089; doi:10.1002/ctm2.70100)
Supplement: Supplementary file 11 — Supporting Information [file CTM2-14-e70100-s014.docx]

**Supplementary Table 2.** Clinical data from control and TBI cases.

| NO. | Group | Age(years) | Gender | BMI | GCS | time post TBI(days) | plasma collected time(days) | depression scales conducted time(days) |
| --- | --- | --- | --- | --- | --- | --- | --- | --- |
| 1 | control | 34 | F | 23.2 | 15 | Not applicable | Not applicable | Not applicable |
| 2 | control | 39 | M | 20.2 | 15 | Not applicable | Not applicable | Not applicable |
| 3 | control | 52 | F | 21.9 | 15 | Not applicable | Not applicable | Not applicable |
| 4 | control | 39 | M | 19.5 | 15 | Not applicable | Not applicable | Not applicable |
| 5 | control | 28 | M | 21.3 | 15 | Not applicable | Not applicable | Not applicable |
| 6 | control | 53 | F | 22.6 | 15 | Not applicable | Not applicable | Not applicable |
| 7 | control | 42 | M | 18.8 | 15 | Not applicable | Not applicable | Not applicable |
| 8 | control | 52 | M | 22.4 | 15 | Not applicable | Not applicable | Not applicable |
| 9 | control | 29 | F | 21.6 | 15 | Not applicable | Not applicable | Not applicable |
| 10 | control | 34 | M | 22.6 | 15 | Not applicable | Not applicable | Not applicable |
| 11 | control | 33 | F | 19.3 | 15 | Not applicable | Not applicable | Not applicable |
| 12 | control | 28 | M | 20.4 | 15 | Not applicable | Not applicable | Not applicable |
| 13 | control | 26 | F | 21.6 | 15 | Not applicable | Not applicable | Not applicable |
| 14 | control | 32 | F | 23.4 | 15 | Not applicable | Not applicable | Not applicable |
| 15 | control | 37 | M | 22 | 15 | Not applicable | Not applicable | Not applicable |
| 16 | control | 43 | F | 23.8 | 15 | Not applicable | Not applicable | Not applicable |
| 17 | control | 21 | F | 21.7 | 15 | Not applicable | Not applicable | Not applicable |
| 18 | control | 22 | M | 19.4 | 15 | Not applicable | Not applicable | Not applicable |
| 19 | control | 34 | F | 22.6 | 15 | Not applicable | Not applicable | Not applicable |
| 20 | control | 42 | M | 19.4 | 15 | Not applicable | Not applicable | Not applicable |
| 21 | control | 31 | F | 21.4 | 15 | Not applicable | Not applicable | Not applicable |
| 22 | control | 34 | M | 23.2 | 15 | Not applicable | Not applicable | Not applicable |
| 23 | control | 50 | F | 19.9 | 15 | Not applicable | Not applicable | Not applicable |
| 24 | control | 37 | M | 20.5 | 15 | Not applicable | Not applicable | Not applicable |
| 25 | control | 30 | M | 22.3 | 15 | Not applicable | Not applicable | Not applicable |
| 26 | control | 55 | M | 23.6 | 15 | Not applicable | Not applicable | Not applicable |
| 27 | control | 44 | F | 21.8 | 15 | Not applicable | Not applicable | Not applicable |
| 28 | control | 56 | M | 21.4 | 15 | Not applicable | Not applicable | Not applicable |
| 29 | control | 32 | M | 19.6 | 15 | Not applicable | Not applicable | Not applicable |
| 30 | control | 38 | F | 18.6 | 15 | Not applicable | Not applicable | Not applicable |
| 31 | control | 42 | F | 19.3 | 15 | Not applicable | Not applicable | Not applicable |
| 32 | control | 43 | M | 22.4 | 15 | Not applicable | Not applicable | Not applicable |
| 33 | control | 28 | F | 19.4 | 15 | Not applicable | Not applicable | Not applicable |
| 34 | control | 34 | M | 21.4 | 15 | Not applicable | Not applicable | Not applicable |
| 35 | control | 36 | F | 23.4 | 15 | Not applicable | Not applicable | Not applicable |
| 36 | control | 42 | F | 18.8 | 15 | Not applicable | Not applicable | Not applicable |
| 37 | control | 20 | F | 19.8 | 15 | Not applicable | Not applicable | Not applicable |
| 38 | control | 28 | M | 24.4 | 15 | Not applicable | Not applicable | Not applicable |
| 39 | control | 38 | F | 21.2 | 15 | Not applicable | Not applicable | Not applicable |
| 40 | control | 46 | M | 17.8 | 15 | Not applicable | Not applicable | Not applicable |
| 41 | TBI | 39 | M | 26.2 | 12 | 90 | 90 | 90 |
| 42 | TBI | 42 | F | 19.6 | 8 | 89 | 89 | 89 |
| 43 | TBI | 50 | M | 19.7 | 10 | 90 | 90 | 90 |
| 44 | TBI | 38 | F | 22.4 | 9 | 91 | 91 | 91 |
| 45 | TBI | 40 | F | 16.4 | 11 | 90 | 90 | 90 |
| 46 | TBI | 38 | F | 19.2 | 9 | 89 | 89 | 89 |
| 47 | TBI | 27 | M | 21.5 | 13 | 91 | 91 | 91 |
| 48 | TBI | 39 | F | 24.6 | 8 | 90 | 90 | 90 |
| 49 | TBI | 42 | M | 21.1 | 11 | 89 | 89 | 89 |
| 50 | TBI | 21 | M | 22.8 | 13 | 90 | 90 | 90 |
| 51 | TBI | 43 | F | 25 | 12 | 90 | 90 | 90 |
| 52 | TBI | 39 | M | 23.2 | 13 | 89 | 89 | 89 |
| 53 | TBI | 52 | F | 22.6 | 8 | 90 | 90 | 90 |
| 54 | TBI | 38 | M | 21.7 | 12 | 91 | 91 | 91 |
| 55 | TBI | 16 | F | 21.3 | 9 | 90 | 90 | 90 |
| 56 | TBI | 21 | M | 23.4 | 11 | 90 | 90 | 90 |
| 57 | TBI | 42 | F | 22.4 | 13 | 90 | 90 | 90 |
| 58 | TBI | 39 | F | 19.4 | 8 | 89 | 89 | 89 |
| 59 | TBI | 41 | M | 22.3 | 12 | 89 | 89 | 89 |
| 60 | TBI | 43 | F | 24.6 | 13 | 90 | 90 | 90 |
| 61 | TBI | 37 | M | 19.6 | 9 | 91 | 91 | 91 |
| 62 | TBI | 40 | M | 22.4 | 12 | 90 | 90 | 90 |
| 63 | TBI | 38 | F | 23.7 | 11 | 89 | 89 | 89 |
| 64 | TBI | 42 | M | 21.7 | 12 | 90 | 90 | 90 |
| 65 | TBI | 29 | F | 22.6 | 10 | 91 | 91 | 91 |
| 66 | TBI | 43 | M | 23 | 12 | 90 | 90 | 90 |
| 67 | TBI | 31 | F | 21.2 | 8 | 89 | 89 | 89 |
| 68 | TBI | 44 | F | 23.6 | 10 | 90 | 90 | 90 |
| 69 | TBI | 29 | F | 20.4 | 13 | 89 | 89 | 89 |
| 70 | TBI | 19 | M | 21.7 | 8 | 90 | 90 | 90 |
| 71 | TBI | 18 | M | 19.4 | 12 | 90 | 90 | 90 |
| 72 | TBI | 43 | F | 16.3 | 13 | 90 | 90 | 90 |
| 73 | TBI | 51 | M | 23.7 | 12 | 90 | 90 | 90 |
| 74 | TBI | 49 | F | 22.9 | 9 | 90 | 90 | 90 |
| 75 | TBI | 37 | F | 25.2 | 11 | 90 | 90 | 90 |
| 76 | TBI | 32 | M | 21.4 | 13 | 90 | 90 | 90 |
| 77 | TBI | 41 | M | 22.6 | 10 | 90 | 90 | 90 |
| 78 | TBI | 37 | F | 21.5 | 12 | 91 | 91 | 91 |
| 79 | TBI | 32 | M | 22.6 | 10 | 90 | 90 | 90 |
| 80 | TBI | 26 | F | 21.8 | 12 | 89 | 89 | 89 |
| 81 | TBI | 42 | M | 24.4 | 13 | 90 | 90 | 90 |
| 82 | TBI | 47 | F | 18.9 | 9 | 91 | 91 | 91 |
| 83 | TBI | 52 | M | 19.3 | 8 | 90 | 90 | 90 |
| 84 | TBI | 39 | F | 22.8 | 11 | 89 | 89 | 89 |
| 85 | TBI | 41 | M | 17.4 | 13 | 90 | 90 | 90 |
| 86 | TBI | 32 | F | 20.2 | 10 | 90 | 90 | 90 |
| 87 | TBI | 26 | M | 22.5 | 12 | 89 | 89 | 89 |
| 88 | TBI | 33 | F | 26.6 | 7 | 90 | 90 | 90 |
| 89 | TBI | 52 | M | 21.4 | 12 | 90 | 90 | 90 |
| 90 | TBI | 56 | F | 23.8 | 10 | 89 | 89 | 89 |
| 91 | TBI | 43 | M | 26 | 13 | 89 | 89 | 89 |
| 92 | TBI | 42 | M | 22.2 | 12 | 90 | 90 | 90 |
| 93 | TBI | 51 | F | 21.6 | 10 | 90 | 90 | 90 |
| 94 | TBI | 39 | F | 23.7 | 13 | 90 | 90 | 90 |
| 95 | TBI | 22 | F | 20.3 | 7 | 91 | 91 | 91 |
| 96 | TBI | 24 | M | 22.4 | 13 | 89 | 89 | 89 |
| 97 | TBI | 47 | M | 21.4 | 12 | 90 | 90 | 90 |
| 98 | TBI | 42 | M | 20.4 | 9 | 90 | 90 | 90 |
| 99 | TBI | 46 | M | 21.3 | 11 | 89 | 89 | 89 |
| 100 | TBI | 48 | F | 25.6 | 12 | 90 | 90 | 90 |

Summary of patient characteristics in control and TBI groups：

| Characteristic | Control Group (Mean ± SD) | TBI Group (Mean ± SD) |
| --- | --- | --- |
| Age (years) | 37.10 ± 9.26 | 38.20 ± 9.37 |
| Gender (Male) | 20:20 | 30:30 |
| BMI | 21.20 ± 1.68 | 21.89 ± 2.20 |
| GCS | 15.00 ± 0.00 | 10.83 ± 1.80 |
| Time Post TBI (days) | Not applicable | 89.80 ± 1.33 |
| Plasma Collected Time (days) | Not applicable | 89.80 ± 1.33 |
| Depression Scales Conducted(days) | Not applicable | 89.80 ± 1.33 |

Note:

GCS: Glasgow Coma Scale
