# Supplementary material for: CircSpna2 attenuates cuproptosis by mediating ubiquitin ligase Keap1 to regulate the Nrf2‐Atp7b signalling axis in depression after traumatic brain injury in a mouse model
Source: Clin Transl Med. 2024 Nov 24;14(11):e70100. doi: 10.1002/ctm2.70100 (PMC11586089; doi:10.1002/ctm2.70100)
Supplement: Supplementary file 12 — Supporting Information [file CTM2-14-e70100-s001.docx]

**Supplementary Table 3.** The primers for qRT-PCR.

| **Target** | **Sequence (5’ → 3’)** |
| --- | --- |
| **circSpna2 (divergent)** | F: GTTAATGAAGTGAGCCAGTTTGC  R: GCTTCTGACGCCGTAAGGTAG |
| **circSpna2 (convergent)** | F: GCTCTCTACCTTACGGCGTC  R: AGCTTTCCCTGCAAGTTGGT |
| ***spna2*** | F: CCTGGAGTCTGAAGGTCTGATG  R: GGAGTTGAAGGTAGCCACTGTATG |
| ***keap1*** | F: TGCCCCTGTGGTCAAAGTG  R: GGTTCGGTTACCGTCCTGC |
| **hsa_circ_0088825** | F: GCGTCGCCGCCACTA  R: ACTTGGGTCCATTTTCGGCT |
| ***atp7b* promoter** | F: CCCCTTAGTGGTGATGACTGG  R: CACATTCTTGGCCTTTTCCCG |
| ***gapdh* (mouse)** | F: AGGTCGGTGTGAACGGATTTG  R: TGTAGACCATGTAGTTGAGGTCA |
| ***GAPDH* (human)** | F: GGAGCGAGATCCCTCCAAAAT  R: GGCTGTTGTCATACTTCTCATGG |
