# Supplementary material for: CircSpna2 attenuates cuproptosis by mediating ubiquitin ligase Keap1 to regulate the Nrf2‐Atp7b signalling axis in depression after traumatic brain injury in a mouse model
Source: Clin Transl Med. 2024 Nov 24;14(11):e70100. doi: 10.1002/ctm2.70100 (PMC11586089; doi:10.1002/ctm2.70100)
Supplement: Supplementary file 13 — Supporting Information [file CTM2-14-e70100-s006.docx]

**Supplementary Table 4**. List of antibodies used in the study

| **Antibody** | **Source** | **Catalog Number** | **Dilution Ratio** | **Species** | **Blocking Serum** |
| --- | --- | --- | --- | --- | --- |
| Nrf2 | Proteintech | 16396-1-AP | 1:1000 | Rabbit | 5% non-fat milk |
| Atp7b | Affinity | AF0410 | 1:1000 | Rabbit | 5% non-fat milk |
| Bdnf | Zenbio | 381133 | 1:1000 | Rabbit | 5% non-fat milk |
| Keap1 | Affinity | AF5266 | 1:1000 | Rabbit | 5% non-fat milk |
| Syn1 | Zenbio | 222757 | 1:2000 | Mouse | 5% non-fat milk |
| β-Actin | GeneTex | GTX109639 | 1:1000 | Rabbit | 5% non-fat milk |
| Lipoic Acid | Abcam | ab58724 | 1:1000 | Rabbit | 5% non-fat milk |
| Lias | Proteintech | 11577-1-AP | 1:1000 | Rabbit | 5% non-fat milk |
| Sdhb | Proteintech | 10620-1-AP | 1:1000 | Rabbit | 5% non-fat milk |
| Fdx1 | Proteintech | 12592-1-AP | 1:1000 | Rabbit | 5% non-fat milk |
| Gapdh | Zenbio | 380626 | 1:1000 | Rabbit | 5% non-fat milk |
