# Supplementary material for: CircSpna2 attenuates cuproptosis by mediating ubiquitin ligase Keap1 to regulate the Nrf2‐Atp7b signalling axis in depression after traumatic brain injury in a mouse model
Source: Clin Transl Med. 2024 Nov 24;14(11):e70100. doi: 10.1002/ctm2.70100 (PMC11586089; doi:10.1002/ctm2.70100)
Supplement: Supplementary file 14 — Supporting Information [file CTM2-14-e70100-s003.docx]

**Supplementary Table 5.**

The sequences of Keap1 and mutants (mut BTB, mut IVR, mut DGR, and mut NTR)

| **Target** | **Sequence (N’ → C’)** |
| --- | --- |
| **Keap1** | MQPEPKLSGAPRSSQFLPLWSKCPEGAGDAVMYASTECKAEVTPSQDGNR TFSYTLEDHT  KQAFGVMNELRLSQQLCDVTLQVKYEDIPAAQFMAHKVVLASSSPVFKAMFTNGLREQ  GMEVVSIEGIHPKVMERLIEFAYTASISVGEKCVLHVMNGAVMYQIDSVVRACSDFLVQQ  LDPSNAIGIANFAEQIGCTELHQRAREYIYMHFGEVAKQEEFFNLSHCQLATLISRDDLNVR  CESEVFHACIDWVKYDCPQRRFYVQALLRAVRCHALTPRFLQTQLQKCEILQADARCKD  YLVQIFQELTLHKPTQAVPCRAPKVGRLIYTAGGYFRQSLSYLEAYNPSNGSWLRLADLQV  PRSGLAGCVVGGLLYAVGGRNNSPDGNTDSSALDCYNPMTNQWSPCASMSVPRNRIGVG  VIDGHIYAVGGSHGCIHHSSVERYEPERDEWHLVAPMLTRRIGVGVAVLNRLLYAVGGFDG  TNRLNSAECYYPERNEWRMITPMNTIRSGAGVCVLHNCIYAAGGYDGQDQLNSVERYDV  ETETWTFVAPMRHHRSALGITVHQGKIYVLGGYDGHTFLDSVECYDPDSDTWSEVTRMT  SGRSGVGVAVTMEPCRKQIDQQNCTC |
| **mut-BTB** | MQPEPKLSGAPRSSQFLPLWSKCPEGAGDAVMYASTECKAEVTPSQDGNR TFSYTLEDHT  KQAFGVMNELRLSQQLCDVTLQVKYEDIPAAQFMAHKVVLASSSPVFKAMFTNGLREQ  GMEVVSIEGIHPKVMERLIEFAYTASISVGKKCVLHVMNGAVMYQIDSVVRACSDFLVQQ  LDPSNAIGIANFAEQIGCTELHQRAREYIYMHFGEVAKQEEFFNLSHCQLATLISRDDLNVR  CESEVFHACIDWVKYDCPQRRFYVQALLRAVRCHALTPRFLQTQLQKCEILQADARCKD  YLVQIFQELTLHKPTQAVPCRAPKVGRLIYTAGGYFRQSLSYLEAYNPSNGSWLRLADLQV  PRSGLAGCVVGGLLYAVGGRNNSPDGNTDSSALDCYNPMTNQWSPCASMSVPRNRIGVG  VIDGHIYAVGGSHGCIHHSSVERYEPERDEWHLVAPMLTRRIGVGVAVLNRLLYAVGGFDG  TNRLNSAECYYPERNEWRMITPMNTIRSGAGVCVLHNCIYAAGGYDGQDQLNSVERYDV  ETETWTFVAPMRHHRSALGITVHQGKIYVLGGYDGHTFLDSVECYDPDSDTWSEVTRMT  SGRSGVGVAVTMEPCRKQIDQQNCTC |
| **mut-IVR** | MQPEPKLSGAPRSSQFLPLWSKCPEGAGDAVMYASTECKAEVTPSQDGNR TFSYTLEDHT  KQAFGVMNELRLSQQLCDVTLQVKYEDIPAAQFMAHKVVLASSSPVFKAMFTNGLREQ  GMEVVSIEGIHPKVMERLIEFAYTASISVGEKCVLHVMNGAVMYQIDSVVRACSDFLVQM  LHCRNAIGIANFAEMIGCTELHQRAREYIYMHFGKVAEMEEFFNLSDCQLFTLISRDDLNV  RCESEVFHACIHWVEYDCPMRRILVMFLLEAVRCHALTPRFLQTQLMECKILQADFECKH  YLVMIFMKLTLHEPTQAVPCRAPKVGRLIYTAGGYFRQSLSYLEAYNPSNGSWLRLADLQ  VPRSGLAGCVVGGLLYAVGGRNNSPDGNTDSSALDCYNPMTNQWSPCASMSVPRNRIGV  GVIDGHIYAVGGSHGCIHHSSVERYEPERDEWHLVAPMLTRRIGVGVAVLNRLLYAVGGFD  GTNRLNSAECYYPERNEWRMITPMNTIRSGAGVCVLHNCIYAAGGYDGQDQLNSVERYD  VETETWTFVAPMRHHRSALGITVHQGKIYVLGGYDGHTFLDSVECYDPDSDTWSEVTRM  TSGRSGVGVAVTMEPCRKQIDQQNCTC |
| **mut-DGR** | MQPEPKLSGAPRSSQFLPLWSKCPEGAGDAVMYASTECKAEVTPSQDGNR TFSYTLEDHT  KQAFGVMNELRLSQQLCDVTLQVKYEDIPAAQFMAHKVVLASSSPVFKAMFTNGLREQ  GMEVVSIEGIHPKVMERLIEFAYTASISVGEKCVLHVMNGAVMYQIDSVVRACSDFLVQQ  LDPSNAIGIANFAEQIGCTELHQRAREYIYMHFGEVAKQEEFFNLSHCQLATLISRDDLNVR  CESEVFHACIDWVKYDCPQRRFYVQALLRAVRCHALTPRFLQTQLQKCEILQADARCKD  YLVQIFQELTLHKPTQAVPCRAPKVGRLIYTAGGYIEQSLSILEAYNPSNGSWLRLADLQVP  RSGLAGCVVGGLLYAVGGRNNSPDGNTDSSALDCYNPMTNQWSPCASMSVPRNRIGVG  VIDGHIYAVGGSHGCIHHSSVERYEPERDEWHLVAPMLTRRIGVGVAVLNRLLYAVGGFDG  TNRLNSAECYYPERNEWRMITPMNTIRSGAGVCVLHNCIYAAGGYDGQDQLNSVERYDV  ETETWTFVAPMRHHRSALGITVHQGKIYVLGGYHGDCIFHSVECYDPDSDTWSKVTEPCQ  YRSGVGVAVTMEPCRKQIDQQNCTC |
| **mut-CTR** | MQPEPKLSGAPRSSQFLPLWSKCPEGAGDAVMYASTECKAEVTPSQDGNR TFSYTLEDHT  KQAFGVMNELRLSQQLCDVTLQVKYEDIPAAQFMAHKVVLASSSPVFKAMFTNGLREQ  GMEVVSIEGIHPKVMERLIEFAYTASISVGEKCVLHVMNGAVMYQIDSVVRACSDFLVQQ  LDPSNAIGIANFAEQIGCTELHQRAREYIYMHFGEVAKQEEFFNLSHCQLATLISRDDLNVR  CESEVFHACIDWVKYDCPQRRFYVQALLRAVRCHALTPRFLQTQLQKCEILQADARCKD  YLVQIFQELTLHKPTQAVPCRAPKVGRLIYTAGGYFRQSLSYLEAYNPSNGSWLRLADLQV  PRSGLAGCVVGGLLYAVGGRNNSPDGNTDSSALDCYNPMTNQWSPCASMSVPRNRIGVG  VIDGHIYAVGGSHGCIHHSSVERYEPERDEWHLVAPMLTRRIGVGVAVLNRLLYAVGGFDG  TNRLNSAECYYPERNEWRMITPMNTIRSGAGVCVLHNCIYAAGGYDGQDQLNSVERYDV  ETETWTFVAPMRHHRSALGITVHQGKIYVLGGYDGHTFLDSVECYDPDSDTWSEVTRMT  SGRSGVGVAVTMEPCREMIDMQNCPQ |
