# Supplementary material for: CircSpna2 attenuates cuproptosis by mediating ubiquitin ligase Keap1 to regulate the Nrf2‐Atp7b signalling axis in depression after traumatic brain injury in a mouse model
Source: Clin Transl Med. 2024 Nov 24;14(11):e70100. doi: 10.1002/ctm2.70100 (PMC11586089; doi:10.1002/ctm2.70100)
Supplement: Supplementary file 15 — Supporting Information [file CTM2-14-e70100-s010.docx]

**Supplementary Table 6.**

The sequences of circSpna2 and mutants (mut1, mut2, mut3, and mut4)

| **Target** | **Sequence (5’ → 3’)** |
| --- | --- |
| **control** | UUUUACCUAGGUUCACCCCAGUUUCACGACCUUUGUCGGCUCCUGUAGGUCCUCUCU  GCUGUCGUUCAGGACCUAGCCAUGGUGGCGAAGUUCCUCGAGAGAUGGAAUGCCGC  AGUCUUCGACCUCCUAAGGAUAGCCAAGGUCAAAAAAGUUUCUCUACGACUCCUCA ACCUCUUCACCUAAGUCCUCUUCGAAGUUCAACGUAGACUACUCUUGAUGUUUCUG  GGUUGGUUGAACGUCCCUUUCGACGUCUUCGUGGUUCGGAAACUUCGACUUCAUGU  CCGAUUGAGUCCUCGGUAACACUUCGACCUGCUCUGUCCUUUGAACUACUAAAGAC  UUCCCGUGAAACGUAGACUUUGGUAGGCCUGUGCAAAUUACCUCGACGUGGCCGUC  ACCCUUGACGAAAACCUCUUCUACGCCCUCUUUCCUUAGUUUGACGACGUCCGUGUC  UUCGACCACGUCAUAAACGCCCUCACACUCCUACAUUACCUGACCUAGUUACUGUUC CUUCGUUAACACUGAAGACUCCUCGACCCGGUCCUGGACCUCGUACAUCUCCACGAU  GUCUUCUUCAAACUUCUCAAAGUCUGACUAGACCGACGAGUACUUCUUUCUCAAUU  ACUUCACUCGGUCAAACGACGGUUUGAGUAGGUC |
| **circSpna2** | AAAAUGGAUCCAAGUGGGGUCAAAGUGCUGGAAACAGCCGAGGACAUCCAGGAGAG  ACGACAGCAAGUCCUGGAUCGGUACCACCGCUUCAAGGAGCUCUCUACCUUACGGCG  UCAGAAGCUGGAGGAUUCCUAUCGGUUCCAGUUUUUUCAAAGAGAUGCUGAGGAGU  UGGAGAAGUGGAUUCAGGAGAAGCUUCAAGUUGCAUCUGAUGAGAACUACAAAGAC  CCAACCAACUUGCAGGGAAAGCUGCAGAAGCACCAAGCCUUUGAAGCUGAAGUACA  GGCUAACUCAGGAGCCAUUGUGAAGCUGGACGAGACAGGAAACUUGAUGAUUUCUG  AAGGGCACUUUGCAUCUGAAACCAUCCGGACACGUUUAAUGGAGCUGCACCGGCAG  UGGGAACUGCUUUUGGAGAAGAUGCGGGAGAAAGGAAUCAAACUGCUGCAGGCACA  GAAGCUGGUGCAGUAUUUGCGGGAGUGUGAGGAUGUAAUGGACUGGAUCAAUGACA  AGGAAGCAAUUGUGACUUCUGAGGAGCUGGGCCAGGACCUGGAGCAUGUAGAGGUG  CUACAGAAGAAGUUUGAAGAGUUUCAGACUGAUCUGGCUGCUCAUGAAGAAAGAGU  UAAUGAAGUGAGCCAGUUUGCUGCCAAACUCAUCCAG |
| **mut1** | AAAAUGGAUCCAAGUGGGGUCAAAGUGCUGGAAACAGCCGAGGACAUCCAGGAGAG  ACGACAGCAAGUCCUGGAUCGGUACCACCGCUUCAAGGAGCUCUCUACCUUACGGCG  UCAGAAGCUGGAGGAUUCCUAUCGGUUCCAGUUUUUUCAAAGAGAUGCUGAGGAGU  UGGAGAAGUGGAUUCAGGAGAAGCUUCAAGUUGCAUCUGAUGAGAACUACAAAGAC  CCAACCAACUUGCAGGGAAAGCUGCAGAAGCACCAAGCCUUUGAAGCUGAAGUACA  GGCUAACUCAGGAGCCAUUGUGAAGCUGGACGAGACAGGAAACUUGAUGAUUUCUG  AAGGCGACUUUGCAUCUGAAACCAUCCGGACACGUUUAAUGGAGCUGCACCGGCAG  UGGGAACUGCUUUUGGAGAAGAUGCGGGAGAAAGGAAUCAAACUGCUGCAGGCACA  GAAGCUGGUGCAGUAUUUGCGGGAGUGUGAGGAUGUAAUGGACUGGAUCAAUGACA  AGGAAGCAAUUGUGACUUCUGAGGAGCUGGGCCAGGACCUGGAGCAUGUAGAGGUG  CUACAGAAGAAGUUUGAAGAGUUUCAGACUGAUCUGGCUGCUCAUGAAGAAAGAGU  UAAUGAAGUGAGCCAGUUUGCUGCCAAACUCAUCCAG |
| **mut2** | AAAAUGGAUCCAAGUGGGGUCAAAGUGCUGGAAACAGCCGAGGACAUCCAGGAGAG  ACGACAGCAAGUCCUGGAUCGGUACCACCGCUUCAAGGAGCUCUCUACCUUACGGCG  UCAGAAGCUGGAGGAUUCCUAUCGGUUCCAGUUUUUUCAAAGAGAUGCUGAGGAGU  UGGAGAAGUGGAUUCAGGAGAAGCUUCAAGUUGCAUCUGAUGAGAACUACAAAGAC  CCAACCAACUUGCAGGGAAAGCUGCAGAAGCACCAAGCCUUUGAAGCUGAAGUACA  GGCUAACUCAGGUCGGAUUGUCUUCCUGGACGAGACAGGAAACUUGUACUUUUCUG  AUCCCCACUUUGCAUCUGAAACCAUCCGGACACGUUUAAUGGAGCUGCACCGGCAG  UGGGAACUGCUUUUGGAGAAGAUGCGGGAGAAAGGAAUCAAACUGCUGCAGGCACA  GAAGCUGGUGCAGUAUUUGCGGGAGUGUGAGGAUGUAAUGGACUGGAUCAAUGACA  AGGAAGCAAUUGUGACUUCUGAGGAGCUGGGCCAGGACCUGGAGCAUGUAGAGGUG  CUACUCUAGAAGUUUGAAGAGUUUCAGACUGUACUGGCUGCAGUUGAAGAAUGAGU  UAAUGAAGUGAGCCAGUUUGCUGCCAAACUCAUCCAG |
| **mut3** | AAAAUGGAUCCAAGUGGGGUCAAAGUGCUGGAAACAGCCGAGGACAUCCAGGAGAG  ACGACAGCAAGUCCUGGAUCGGUACCACCGCUUCAAGGAGCUCUCUACCUUACGGCG  UCAGAAGCUGGAGGAUUCCUAUCGGUUCCAGUUUUUUCAAAGAGAUGCUGAGGAGU  UGGAGAAGUGGAUUCAGGAGAAGCUUCAAGUUGCAUCUGAUGAGAACUACAAAGAC  CCAACCAACUUGCAGGGAAAGCUGCAGAAGCACCAAGCCUUUGAAGCUGAAGUACA  GGCUAACUCAGGAGCCAUUGUGAAGCUGGACGAGACAGGAAACUUGAUGAUUUCUG  AAGGGCACUUUGCAUCUGAAACCAUCCGGACACGUUUAAUGGAGCUGCACCGGCAG  UGGGAACUGCUUUUGGAGAAGAUGCGGGAGAAAGGAAUCAAACUGCUGCAGGCACA  GAAGCUGGUGCAGUAUUUGCGGGAGUGUGAGGAUGUAAUGGACUGGAUCAAUGACA  ACCUUGCAAUUGUGACUUCACAGGAGCUGGGCCAGGACCUGGAGCAUGUAGAGGUG  CUACAGAAGAAGUUUGAAGAGUUUCAGACUGAUCUGGCUGCUCAUGAAGAAAGAGU  UAAUGAAGUGAGCCAGUUUGCUGCCAAACUCAUCCA |
| **mut4** | AAAAUGGAUCCAAGUGGGGUCAAAGUGCUGGAAACAGCCGAGGACAUCCAGGAGAG  ACGACAGCAAGUCCUGGAUCGGUACCACCGCUUCAAGGAGCUCUCUACCUUACGGCG  UCAGAAGCUGGAGGAUUCCUAUCGGUUCCAGUUUUUUCAAAGAGAUGCUGAGGAGU  UGGAGAAGUGGAUUCAGGAGAAGCUUCAAGUUGCAUCUGAUGAGAACUACAAAGAC  CCAACCAACUUGCAGGGAAAGCUGCAGAAGCACCAAGCCUUUGAAGCUGAAGUACA  GGCUAACUCAGGAGCCAUUGUGAAGCUGGACGAGACAGGAAACUUGAUGAUUUCUG  AAGGGCACUUUGCAUCUGAAACCAUCCGGACACGUUUAAUGGAGCUGCACCGGCAG  UGGGAACUGCUUUUGGAGAAGAUGCGGGAGAAAGGAAUCAAACUGCUGCAGGCACA  GAAGCUGGUGCAGUAUUUGCGGGAGUGUGAGGAUGUAAUGGACUGGAUGAAUGAC  AAGGAAGCAAUUGUGACUUCUGAGGAGCUGCCGCAGGACCUGGAGCAUGUAGUCCU  GCUACAGAAGAAGUUUGAAGAGUUUCAGACUGAUCUGGCUGCUCAUGAAGAAAGAG  UUAAUGAAGUGAGCCAGUUUGCUGCCAAACUCAUCCAG |
